# The Evolution of the First Code

**DOI:** 10.3390/genes17050544

**Published:** 2026-05-02

**Authors:** Lei Lei, Savio Torres de Farias, Zachary Frome Burton

**Affiliations:** 1 School of Biological Sciences, University of New England, Biddeford, ME 04005, USA; llei@une.edu; 2 Laboratório de Genética Evolutiva Paulo Leminski, Departamento de Biologia Molecular, Universidade Federal da Paraíba, João Pessoa 58051-900, PB, Brazil; stfarias@yahoo.com.br; 3 Department of Biochemistry and Molecular Biology, Michigan State University, East Lansing, MI 48824-1319, USA

**Keywords:** genetic code, tRNA, aminoacyl-tRNA synthetase, tRNA modifications, network analyses, last universal common (cellular) ancestor, tRNA-linked chemistry, abiogenesis, astrobiology

## Abstract

**Background/Objectives**: tRNAs, tRNAomes, aminoacyl-tRNA synthetases (AARSs), the first proteins, ribosomes and the genetic code coevolved. We utilize sequence data to reconstruct key steps in establishing the first code on Earth. **Methods**: Networks were constructed to describe initial tRNAome and AARSome evolution. **Results**: tRNA-34 wobble and tRNA-37 modifications were necessary to evolve the code, as were additional tRNA modifications, so diverse tRNA modification enzymes (i.e., histidyl-tRNA -1 GTP synthase) are among the first proteins. tRNA-linked chemistry brought asparagine, glutamine, cysteine and possibly additional amino acids into the code. tRNA, tRNA modifications and tRNA-linked chemistry were core founding innovations for code evolution. Coevolution of AARSomes was also essential. Class II and class I AARSs have distinct folds but are nonetheless homologs by sequence. Early AARS enzymes folded around Zn motifs. Networks were generated for tRNAomes and AARSomes in ancient Archaea, because Archaea are the closest living organisms to the last universal common ancestor. **Conclusions**: The first code on Earth was surprisingly ordered, and the few apparent deviations from the regular order can yet be explained. Early in the evolution of the code, innovation was more strongly selected than accuracy. The code froze, however, because of evolving fidelity mechanisms. A historical record was documented in tRNA and in the genetic code structure and has been preserved in living organism sequences. AARSome structure describes the first code evolution more adequately than tRNAomes.

## 1. Introduction

To evolve complex life requires a genetic code, which requires a genetic adapter. Without a code supported by an adapter molecule, the potential to evolve enduring and replicated complexity based on pre-life metabolic systems remained limited [1,2,3,4]. Life on Earth evolved around tRNA, tRNAomes, AARSomes, first proteins, ribosomes and the genetic code [5,6,7,8,9,10]. The purpose of this review is to concentrate on early tRNAome and AARSome networks to describe the evolution of the first code on Earth.

For evolution of the code, tRNAs must diversify to tRNAomes. Most tRNAs are type I, initially, with a 5 nt V loop (V for variable). In Archaea, longer type II V arms (initially 14 nt) are utilized by tRNA^Leu^ (5 tRNA^Leu^) and tRNA^Ser^ (4 tRNA^Ser^). The type I V loop was processed from the primitive type II V arm by a 9 nt internal deletion [11]. Leucine and serine occupy 6-codon sectors of the code, so their longer V arms were used in place of the anticodon stem–loop–stem as a major determinant for cognate AARS recognition. Arginine is also found in a 6-codon sector of the code (5 tRNA^Arg^). Arginine utilizes significant anticodon loop unwinding to expose additional bases for recognition. It is not likely that the strategy utilized for arginine could support three amino acids in 6-codon boxes. Anticodon loop unwinding indicates allosteric effects of cognate tRNA-AARS binding [11,12,13,14]. Complex life on Earth evolved around tRNA, tRNAomes and AARSomes.

AARSomes diverged from class II to class I enzymes [15,16,17,18]. GlyRS-IIA (class II; subclass A) appears to be closest to the founding AARS. It follows that glycine was the founding amino acid in the code [19,20]. All class II enzymes were derived from GlyRS-IIA as the root sequence. A primitive ValRS-IA (class I; subclass A) was derived from a primitive GlyRS-IIA by appending an N-terminal extension, which redirected to the class I AARS fold. Early folds of class II and class I AARS were directed by Zn binding. All class I AARS appear derived from a primordial ValRS-IA as the root enzyme. AARS enzymes are analyzed for: (1) tRNA contacts; (2) tRNA deformation (allostery); (3) modifications of the anticodon loop; (4) amino acid identity (chemical features); and (5) fidelity (i.e., editing/proofreading). These characteristics appear to be most central to the establishment of the first code. At early stages, code innovation was more important than fidelity. At late stages, fidelity mechanisms froze the code.

We utilize the ancient Archaeon *Pyrococcus furiosus* as a reference species that may be close to the last universal common (cellular) ancestor (LUCA) for translation functions [21]. The *P. furiosus* tRNAome is tightly clustered around the primordial tRNA sequence. Similarly, the AARSome appears to be diverged in an orderly manner from the primitive GlyRS-IIA root sequence. Of course, tRNAomes and AARSomes must diverge from root sequences to maintain cognate translational discrimination and accuracy.

As described in analyses of tRNAome and AARSome sequences, the evolution of tRNA and the genetic code fits naturally with the amyloid hypothesis for the origin of life [22,23]. We imagine a complex ribozyme, co-factor, tRNA–amino acid, tRNA–peptide, metabolism, polyglycine, polyGADV, amyloid, coacervate, protocell world coevolved to emulsify and form the first cells [24,25,26,27,28,29]. According to our understanding, an RNA world lacking complementary components is an oversimplification. Analyses of tRNA sequences and evolutionary history reveal a complex pre-life world with substantial chemical and metabolic capacities. The first proteins that coevolved with the code were long, complex and specialized functions [15,30,31].

## 2. Materials and Methods

Based on our previous analyses, *P. furiosus* was the reference species chosen to be similar to LUCA for translation functions [21]. In the future, selecting a more advantageous reference species that is closer to LUCA may be possible and may provide additional insights. The idea behind the choice of *P. furiosus* was to anchor our research to a system lacking huge divergence from the first code. The root sequence for tRNA evolution has been determined and essentially matches a typical tRNA from *P. furiosus* [31]. Defining or estimating root sequences is fundamental to understanding early evolution and the pre-life-to-life transition. It appears that a primitive GlyRS-IIA diversified to all class II AARSs. A primitive GlyRS-IIA apparently diverged to a primitive ValRS-IA by attachment of an N-terminal segment that redirected the protein fold [15]. All class I AARSs appear to diverge from a primitive ValRS-IA.

Sequence similarity of class II and class I AARSs has been demonstrated. For instance, the sequence similarity of *Methanobacterium bryantii* IleRS-IA (a class I AARS) and *Methanococcoides burtonii* GlyRS-IIA (a class II AARS) was determined with an e-value of 4 × 10^−12^ for a substantial in-phase alignment [15,18]. The e-value represents about 1 chance in 2.5 × 10^11^ of the alignment resulting from a random occurrence. Many more examples of class II versus class I AARS homology can readily be obtained. These data are inconsistent with some other published models for class II and class I AARS evolution [32,33,34,35]. The Carter–Ohno–Rodin model indicated that class I and class II AARSs evolved separately, each from a complementary strand of a short ancestral bidirectional gene. In their model, small class I and class II AARS “urzymes” expanded to modern AARS enzymes. Because class II and class I AARS enzymes are linear, direct homologs by sequence, the Carter–Ohno–Rodin model cannot be correct. Also, class II and class I AARSs were complex enzymes and not urzymes, being first enzymes that coevolved with the genetic code.

A 2-dimensional network for *P. furiosus* AARS enzymes was previously published [18]. Phyre 2 scoring of structural and sequence similarity was used to draw maps for class II and class I AARSs separately. Because high homology scores in Phyre 2 were assigned to closely related enzymes, reciprocal scores were used to draw the maps. At the time the maps were constructed, there was no objective mechanism to incorporate the sequence similarity of GlyRS-IIA, IleRS-IA and ValRS-IA. Phyre 2 could not do or score this alignment because of the distinct class IIA and class IA folds.

AARS enzymes utilize editing reactions in their aminoacylating active site (i.e., ValRS-IA, MetRS-IA, IleRS-IA, LeuRS-IA, AlaRS-IID, ThrRS-IIA, ProRS-IIA and SerRS-IIA) that blocks the incorporation of non-cognate amino acids. In separate proofreading active sites (i.e., ValRS-IA, IleRS-IA, LeuRS-IA, PheRS-IIC, AlaRS-IID and ThrRS-IIA), some AARSs remove non-cognate amino acids after attachment to a cognate tRNA [36,37]. Editing and proofreading assays were done in a small number of reference organisms, so results may not universally apply.

Structural studies also tend to be done in a small number of reference organisms. ChimeraX (version 1.10) was used to generate molecular graphics [38,39,40]. AARS structures were selected that were close to the first enzymes. AARS structures were drawn to describe the most important tRNA contacts and deformations and are not meant to be a complete description.

The Modomics database was used to identify anticodon loop modifications [41]. *P. furiosus* anticodon loop modification data were obtained from reference [42].

tRNA structures were drawn and colored according to internal homologies, based on the three 31 nt minihelix tRNA evolution theorem, as previously described [15,30,31]. Historical numbering of tRNAs can be confusing, particularly within the D loop and V loop. Here, we number the D loop D_1_ to D_17_ and the V loop V_1_ to V_n_ (V loop of n bases; initially, V_1_ to V_5_ for type I and V_1_ to V_14_ for type II; V_1_ to V_5_ for type I align with V_1_ to V_5_ for type II). Type I V loops and type II V arms have been misaligned in tRNA databases.

## 3. Evolution of tRNA

**Figure 1:**
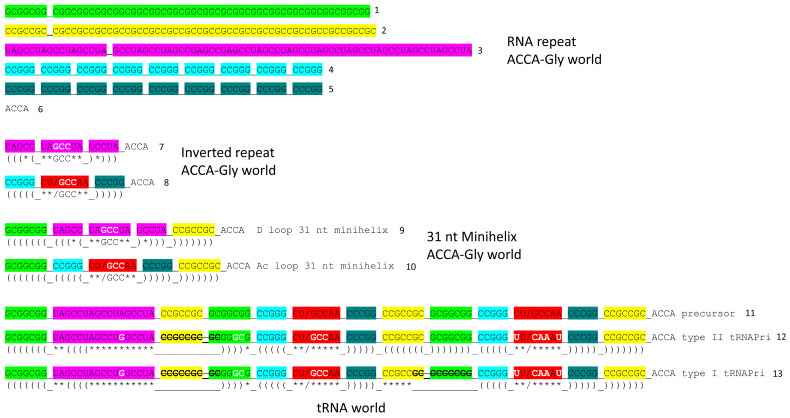
tRNA evolved from RNA repeats and inverted repeats (stem–loop–stems) of known and conserved sequence. See the text for details. ACCA-Gly (line 6) was the primordial adapter molecule. Colors reflect internal homologies and are consistent throughout the figures. The tRNA precursor (line 11) was generated by the ligation of a 31 nt D loop minihelix (line 9, lacking 3′-ACCA) and two 31 nt anticodon (Ac) minihelices (line 10, lacking 3′-ACCA). Internally deleted bases are indicated in bold with strike-throughs. Bases in white bold are anticodon bases or sequence changes to support the tRNA fold. During pre-life, RNAs were formed with and without ligated 3′-ACCA. Underscored positions separate sequence elements and indicate how stem and loop sequences were selected. We posit that GCC may have been the primordial anticodon. GCC can utilize a GGC repeat (line 1) as a pre-mRNA. Secondary structures are indicated (parentheses stand for stems; * stands for loops).

**Figure 2:**
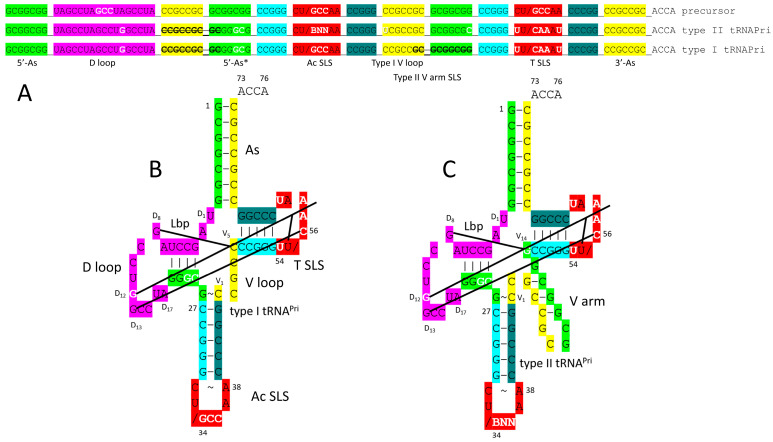
The tRNA fold caused selection of a small number of systematic sequence changes compared to the tRNA precursor. (**A**) A linear comparison of the tRNA precursor, type II tRNA^Pri^ and type I tRNA^Pri^. (**B**) Type I tRNA^Pri^. (**C**) Type II tRNA^Pri^. B indicates G, C or U, not A (A is not utilized in the wobble tRNA-34 position in Archaea). Black lines indicate the Levitt base pair (Lbp) and elbow contacts (where the D loop binds the T loop). White bold bases are the anticodon and systematic sequence changes before LUCA to support the tRNA fold. As) stands for acceptor stems; Ac) stands for anticodons; SLS) stands for stem–loop–stems.

The evolution of tRNA has been solved to the last nucleotide (Figure 1 and Figure 2) [15,30,31]. The sequences of tRNAs in living organisms validate the model. In Figure 1, the linear sequences that formed tRNAs are shown. In Figure 2, the folding of the linear tRNA sequence is indicated. tRNA evolved from 100% RNA repeats and inverted repeats of known sequence plus 3′-ACCA (Figure 1; lines 1–8). ACCA was the primordial adapter molecule. In pre-life, ACCA-Gly was ligated to numerous RNAs to synthesize polyglycine. Multiple ACCA-Gly (line 6) paired with an extended GCGGCGGCG repeat (line 1) are capable of synthesizing polyglycine using wet–dry cycling and published procedures [43]. tRNAomes in ancient Archaea validate the model for tRNA evolution to such an extent that the model is referred to as a theorem (a proven theory). A typical tRNA from an ancient Archaeon has the same sequence as is shown (Figure 1; lines 12 and 13) [15,30,31]. Sequence logos demonstrate the theorem. tRNA and 31 nt minihelices evolved initially as improved mechanisms to synthesize polyglycine. tRNA evolved from the ligation of three 31 nt minihelices (one D loop minihelix (line 9) and two anticodon (Ac) stem–loop–stem minihelices (line 10)). The ligation of minihelices formed long RNAs from which segments were removed by endo- and exo-nucleases, selecting RNA stem lengths and stem–loop–stems. In tRNA, the 17 nt Ac stem–loop–stem and the 17 nt T stem–loop–stem are homologs, and obviously so, just from inspection. By contrast, the 31 nt D loop minihelix had a 17 nt UAGCC repeat core (D_1_-UAGCCUAGCCUAGCCUA-D_17_) (line 7). The small number of sequence changes from the primordial sequence, indicated in white bold, were selected to support the tRNA fold.

Figure 2 shows how the tRNA fold caused selection of a small number of changes in the primordial tRNA precursor sequence. The tRNA precursor was a replication intermediate for the 31 nt minihelix world. tRNA was generated by a folding and processing “error”. Type II tRNA was generated from the precursor by a single internal 9 nt deletion within ligated 3′- and 5′-acceptor stems. Type I tRNA was generated by an additional internal 9 nt deletion within ligated 3′- and 5′-acceptor stems, within the V arm region, so a primitive type II tRNA could have been processed by an internal 9 nt deletion to type I tRNA. The two 9 nt internal deletions to form type I tRNA were identical on complementary strands.

There are alternative published views of tRNA evolution, but they cannot be correct because they are falsified by existing tRNA sequences. No accretion model, convergent model or two minihelix model can possibly be correct [44,45,46,47,48,49,50,51]. Clearly, the 17 nt anticodon stem–loop–stem and the 17 nt T stem–loop–stem of tRNA are homologous sequences [15,30,31]. No two minihelix model, convergent model or accretion model is consistent with this obvious homology. Just as clearly, the 17 nt D loop minihelix core is based on a UAGCC repeat. Many D loops in ancient Archaea have two perfect UAGCC repeats [52,53,54]. Acceptor stems and acceptor stem remnants (i.e., type II V arms and type I V loops) are based on GCG and CGC repeats. tRNAomes diverged from a single root tRNA sequence. The three 31 nt minihelix model made and fulfilled predictions: (1) tRNA sequences in ancient Archaea and their radiations to tRNAomes can be understood; (2) sequence logos support the model; (3) statistical evaluations of sequences support the model; (4) primordial tRNA sequences have been confirmed; (5) proper alignments can be adjusted for D loops and V loops; and (6) the proper alignment of type II and type I V arms and V loops has been obtained (a type I V loop was derived from a 9 nt deletion within a 14 nt type II V arm) [11,15,30,31] (Figure 1 and Figure 2). A tRNA sequence has been traced to its root in pre-life. Tracing root sequences to pre-life strongly supports tRNA as the primordial function around which translation systems and the genetic code evolved. If the three 31 nt minihelix tRNA evolution theorem is accepted and applied, tRNA evolution and the evolution of the genetic code can be understood. If the theorem is rejected, no adequate description of tRNA evolution or of the genetic code can be reasonably inferred. All two minihelix and accretion models are random sequence models that cannot be supported or rationalized using sequences of ancient Archaeon species.

## 4. AARS Enzymes at the Base of Code Evolution

### 4.1. The AARS Mechanism

The AARS enzyme reaction is complex [36,37,55]. Within the aminoacylating active site of AARSs, the amino acid carboxy terminus reacts with ATP to form an AMP adduct (aa-C=O, -O–AMP), releasing pyrophosphate. The tRNA 73-NCCA (N is the discriminator base) end displaces AMP to bind the aa-C=O, -O-tRNA, releasing AMP. Class II AARSs attach the 76A ribose 3′-O to the cognate amino acid. Class I AARSs attach the 76A ribose 2′-O to the cognate amino acid. ATP, the cognate amino acid and the cognate tRNA are substrates. Because the reaction progresses in two steps, the order of substrate additions may be important for AARS enzymes, affecting whatever reaction intermediate analogues or AARS-tRNA conformations can be visualized in crystal or cryo-electron microscopy structures. Pyrophosphate, AMP and aa-tRNA are products. In structures, non-reactive aa-AMP analogues were sometimes used to mimic a reaction intermediate.

### 4.2. GlyRS-IIA

**Figure 3:**
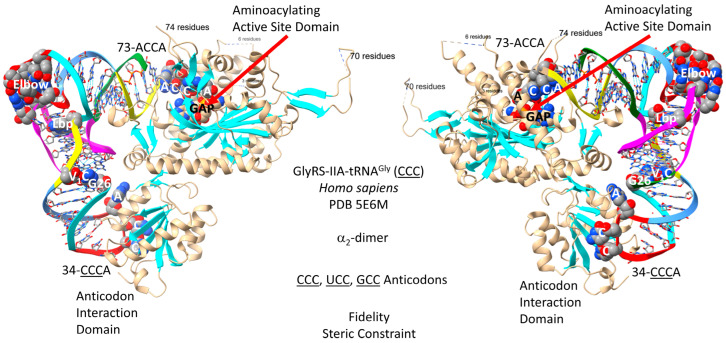
GlyRS-IIA-tRNA^Gly^ (CCC) from *H. sapiens*. A primitive GlyRS-IIA appears to be the founding AARS. This image was selected to emphasize tRNA contacts. Proteins are in beige. β-sheets are in cyan. tRNAs are colored according to the three 31 nt minihelix tRNA evolution theorem (Figure 1 and Figure 2). Some GlyRS-IIA amino acids that were not imaged are noted. Lbp stands for the Levitt base pair. The elbow is where the D loop binds the T loop.

A primordial GlyRS-IIA appears to be the founding AARS. All class II and class I AARS enzymes appear to be derived from this root. Figure 3 shows a GlyRS-IIA-tRNA^Gly^ (CCC) structure from *Homo sapiens* [56]. Human GlyRS-IIA is similar in structure and sequence to archaeal GlyRS-IIA. GlyRS-IIA is an α_2_-dimer, but the image is of only a single GlyRS-IIA-tRNA^Gly^ (CCC). The image was selected to demonstrate primary tRNA^Gly^ contacts to the anticodon loop and the tRNA 73-ACCA-76 3′-end. As a class II AARS, GlyRS-IIA has its aminoacylating active site on a surface of antiparallel β-sheets. The GAP reaction intermediate analogue and the 73-ACCA-76 sequence identify the aminoacylating active site. tRNA^Gly^ utilizes 34-CCC-36, UCC and GCC anticodons (anticodons are underlined for clarity), so the strongest interactions with GlyRS-IIA might be 35-CCA-37, as indicated in the structure.

**Figure 4:**
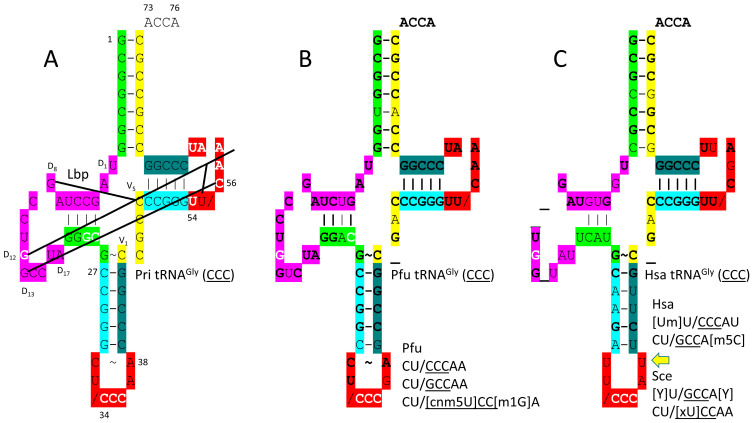
tRNA^Gly^ (CCC). (**A**) A primordial tRNA^Gly^ (CCC). (**B**) *P. furiosus* tRNA^Gly^ (CCC). (**C**) Human tRNA^Gly^ (CCC). tRNAs are colored according to the three 31 nt minihelix tRNA evolution theorem (Figure 1 and Figure 2) [15,31]. In Figure 4A, the Levitt base pair (D_8_G = V_5_C) (Lbp) and some elbow contacts are indicated. The Levitt base pair is a reverse Watson–Crick pair that forms two hydrogen bonds. D_12_G intercalates between 57A and 58A and hydrogen bonds to 55U [60]. D_13_G forms a Watson–Crick pair with T loop 56C. Anticodon sequences are underlined or in white bold. / indicates a U-turn. Modifications of the anticodon loop are indicated in the sequences. Modomics notation is used for tRNA anticodon loop modifications [41]. xU indicates an unknown 5-carbon U modification to suppress superwobbling. Yellow arrows indicate features that may be of interest. 32U-38U is expected to alter dynamics of the anticodon loop.

Figure 4 shows tRNA^Gly^ (CCC). Figure 4A is the primordial tRNA^Gly^ (CCC). Figure 4B is the *P. furiosus* tRNA^Gly^ (CCC). Figure 4C is the human (Hsa) tRNA^Gly^ (CCC), as in the structure in Figure 3 [41,54,57,58]. Modifications to the anticodon loop are indicated and explained in the figure legend. Conserved bases compared to the primordial tRNA^Gly^ are in bold in Figure 4B,C. As previously described, the primordial tRNA^Gly^ (CCC) is a highly ordered sequence formed from GCG (5′-acceptor stem and 5′-acceptor stem remnant (5′-As*)), CGC (3′-acceptor stem and 3′-acceptor stem remnant (type I V loop)), and UAGCC (D loop) repeats and inverted repeats (stem–loop–stem; ~CCGGG_CU/CCCAA_CCCGG; _ indicates separation of stem and loop; / indicates a U-turn; the anticodon is underlined) [15,31]. A few deviations from the perfectly ordered initial sequence are noted (Figure 4A). These deviations, which pre-date LUCA, support the tRNA fold. D_12_G (replacing D_12_A in the third UAGCC repeat) intercalates between 57A and 58A and hydrogen bonds to 55U. D_13_G forms a Watson–Crick pair with 56C. These are referred to as “elbow” contacts, where the D loop binds the T loop to stabilize the tRNA form [15,59]. The T loop is strongly selected to obtain the typical sequence UU/CAAAU to maintain the interaction with the D loop at the elbow.

In *P. furiosus,* tRNA^Gly^ is the most similar tRNA to tRNA^Pri^ (Pri stands for primordial) [21,41]. The acceptor stem matches the primordial sequence in all but 1 bp. The primordial acceptor stem sequence is matched perfectly in some Archaea (i.e., *Staphylothermus marinus* tRNA^Gly^ (GCC); GCGGCGG; a GCG repeat) [52]. In tRNA^Gly^ (CCC) of *P. furiosus*, the D loop is intact (D_1_-UAGUCUAGCCUGGUCUA-D_17_; Figure 4B) and very similar to tRNA^Pri^ (D_1_-UAGCCUAGCCUGGCCUA-D_17_; Figure 4A), with only two base changes from the primordial sequence and no deleted bases relative to tRNA^Pri^. The 5′-As* sequence GGACG varies in only a single base from the typical primordial sequence GGGCG. The anticodon stem matches tRNA^Pri^ in 4 of 5 bp. The V loop sequence C_GAC matches the primordial sequence CCGCC in 3 of 5 positions. The T stem–loop–stem matches tRNA^Pri^ at every position. As expected, human tRNA^Gly^ is more innovated from tRNA^Pri^, but tRNA^Gly^ (CCC) in humans is so similar that it might be monophyletic with tRNA^Gly^ (CCC) in Archaea such as *P. furiosus*.

In *P. furiosus*, only the 34U anticodon loop for tRNA^Gly^ appears to be modified [42]. The 34cnm5U modification is initiated by Elp3, which is a first enzyme that is as ancient as the genetic code. A 34U modified at the 5-carbon position is expected to limit superwobbling. Unmodified 34U can read the codon wobble 3-A,G,C and U. In mitochondria, superwobbling is utilized in 4-codon boxes to shrink the size of the tRNAome [61,62,63]. A single unmodified 34U tRNA can read an entire 4-codon box. Because glycine is in a 4-codon box, unmodified 34U might be tolerated, but the 34cnm5U modification would limit reading to codons 3-A and 3-G.

Anticodon loops 32C-38A form a hydrogen bond [60]. A 32Y-38Y (Y stands for pseudouridine), 32Um-38U, or 32C-38m5C interaction would be expected to change the dynamics of the loop.

Glycine is the smallest and most flexible amino acid. Steric hindrance of larger amino acids may be a mechanism by which GlyRS-IIA limits misincorporation of non-cognate amino acids. In more innovated Bacteria (i.e., *Escherichia coli*), GlyRS-IID substitutes for GlyRS-IIA. GlyRS-IIA is the more ancient enzyme and appears to be the root of both the class II and class I AARS lineages [16,18,64].

The genetic code is hypothesized to have initially evolved to synthesize polyglycine, making tRNA^Gly^ the first tRNA, and a primitive GlyRS-IIA appears to be the founding AARS, as indicated by sequence alignment. In pre-life, single-stranded RNA may have been stabilized by methylation at the 2′-O [43]. This modification would render RNA resistant to ribozyme ribonucleases and base hydrolysis. If the 2′-O were modified in pre-life (i.e., by methylation), this might explain why GlyRS-IIA initially evolved to utilize the tRNA-76 ribose 3′-O to attach glycine.

### 4.3. ValRS-IA

The founding class I AARS appears to be a primitive version of ValRS-IA [16,17,18,64]. All class I AARSs appear to radiate from this root. A primitive ValRS-IA was derived from a primitive GlyRS-IIA by attachment of an N-terminal sequence that redirected to the distinct class I fold. In ancient Archaea, ValRS-IA and IleRS-IA have two Zn motifs: one in the added N-terminal segment, and one in the segment that is homologous to the N-terminal Zn motif in GlyRS-IIA. The added N-terminal Zn motif to form class I AARSs generates the class I fold. It appears that early folding of class II and class I AARSs was highly dependent on these Zn motifs. As evolution progressed, the Zn motifs were sometimes replaced by other folding determinants. Because AARSs are the first proteins (coevolved with the genetic code), early folding mechanisms dependent on Zn indicate early entry of cysteine into the code.

Glycine, alanine, aspartic acid and valine (GADV) are proposed to be the first encoded amino acids [65,66,67,68]. GADV (the four simplest amino acids) are located at the 4th row of the code (tRNA-36C). The genetic code appears to have been sectored primarily by code columns. It is hypothesized that the evolution of columns commenced by filling the code with GADV in the favored row 4 and, perhaps, expanding into other rows. It is hypothesized that earlier encoded amino acids occupied larger segments of the code that were then invaded by incoming amino acids. Amino acids that were added early subsequently retreated to occupy the most favored sectors of the code (i.e., tRNA-36C). Glycine is located at code column 4 (tRNA-35C). Alanine is located at code column 2 (tRNA-35G). Aspartic acid is located at code column 3 (tRNA-35U). Valine is located at code column 1 (tRNA-35A). It is hypothesized that row 4 (tRNA-36C) and column 4 (tRNA-35C) were the most favored in establishing the code. As the first encoded amino acid, glycine occupies the most favored row and column in the code. The genetic code is a highly ordered assembly.

Valine appears to be the founding amino acid for the assembly of column 1 of the code (tRNA-35A). Assembly of column 1 can be considered from the points of view of similar amino acids and homologous AARS enzymes. As an order of assembly, in column 1, we posit that V (row 4) evolved to L (rows 1 and 2), which evolved to I (row 3), which evolved to M (row 3). F appears to be a later addition to column 1, row 1, in the disfavored row 1. According to closely homologous AARS enzymes, consider the following order of evolution: ValRS-IA evolved to LeuRS-IA, which evolved to IleRS-IA, which evolved to MetRS-IA. The entry of phenylalanine and PheRS-IIC will be discussed below. The disfavored row 1 (tRNA-36A) appears to fill last and is a separate case. Sequence preference for rows appears to follow the following order: C (row 4; tRNA-36C) was favored over G (row 2; tRNA-36G), which was favored over U (row 3; tRNA-36U), which was strongly favored over A (row 1; tRNA-36A). In row 3, it appears that Met invaded an Ile 4-codon sector, eliminating the UAU anticodon and inducing differential tRNA-34 modifications of CAU to discriminate Ile (GAU and agm2CAU) (agm2C for agmatidine) and Met (CAU (initiator) and CmAU (elongator). Modification of the 2-carbon of C (agm2C) (Ile) slightly resembles G (Ile) and discriminates from Met (2-carbon C=O).

**Figure 5:**
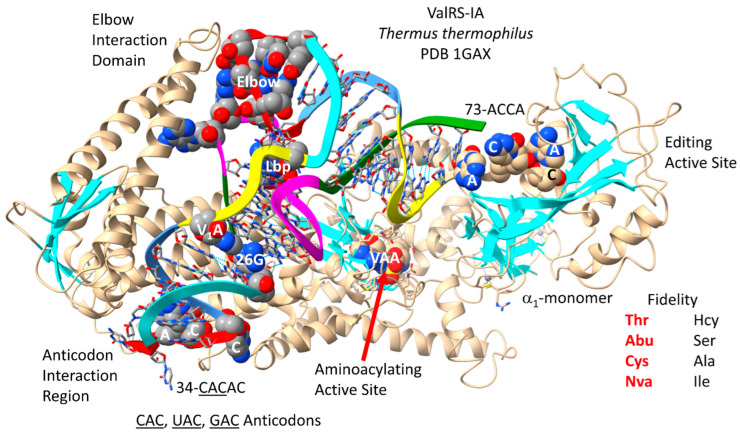
ValRS-IA-tRNA^Val^ (CAC) from *T. thermophilus*. The color scheme used in this image is the same as that in Figure 3. Non-cognate amino acids that are blocked from incorporation within the aminoacylating active site are indicated in red. Non-cognate amino acids that are removed from tRNA^Val^ after attachment within the separate proofreading (editing) active site are indicated in black.

The ValRS-IA of *Thermus thermophilus* bound to tRNA^Val^ (CAC) is shown in Figure 5 [69]. ValRS-IA functions as an α_1_-monomer. Because ValRS-IA is a class I AARS, the aminoacylating active site is at the C-terminal ends of a set of parallel β-sheets. The arrangement of parallel β-sheets has been described as a Rossmann fold, but, undoubtedly, the aminoacylating active site arrangement of ValRS-IA and other class I AARSs is genetically unrelated to Rossmann fold proteins. The aminoacylating active site can also be identified by the binding of VAA, a non-reactive Val-AMP analogue. 73-ACCA-76 is located at the separate editing active site that removes non-cognate amino acids from tRNA^Val^. Non-cognate homocysteine, serine, alanine and isoleucine can be removed by the separate proofreading (editing) active site after attachment to tRNA^Val^. Reactions within the ValRS-IA aminoacylating active site limit non-cognate threonine, α-aminobutyric acid, cysteine and norvaline attachments to tRNA^Val^. As a small, hydrophobic amino acid, valine has little chemical character, so editing reactions both before and after tRNA^Val^ attachment are important to limit inaccurate translation [36,37].

**Figure 6:**
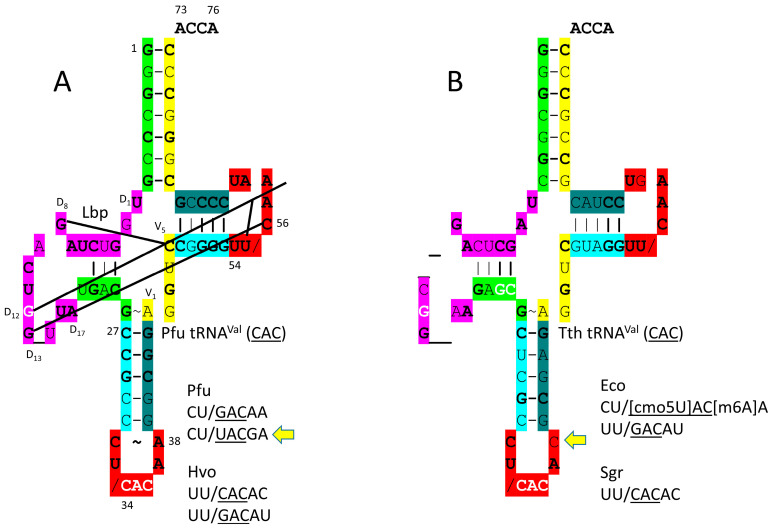
tRNA^Val^ (CAC). (**A**) From *P. furiosus* (Pfu). The yellow arrow indicates an unmodified 34U. (**B**) From *T. thermophilus* (Tth). The yellow arrow indicates a 32C-38C arrangement, which may affect loop dynamics. Hvo indicates *Haloferax volcanii*. Eco indicates *E. coli*. Sgr indicates *Streptomyces griseus*.

tRNA^Val^ is shown in Figure 6. In Figure 6A, *P. furiosus* tRNA^Val^ (CAC) is shown. The acceptor stem matches the primordial tRNA sequence in 4 of 7 bp. The D loop has the sequence D_1_-UGGUCUAGACUGG_UUA-D_17_ and matches the primordial sequence in all but five positions, with a single base deleted from tRNA^Pri^. The anticodon stem matches the primordial sequence in 3 of 5 bp. The T stem–loop–stem matches the primordial sequence in all but 1 stem bp. In *P. furiosus*, tRNA^Val^ is similar to tRNA^Ala^ in sequence, indicating that Val and Ala may have entered the code at about the same time in evolution. GADV are proposed to have been the first four encoded amino acids [65,66,67]. The *T. thermophilus* tRNA^Val^ (CAC) is more derived from the root sequence, as expected (Figure 6B). Bacteria are more derived from LUCA than Archaea.

tRNA^Val^ utilizes CAC, UAC and GAC anticodons. In *P. furiosus* and *H. volcanii*, none of these anticodon loops appear to be modified [42,70]. Unmodified UAC would be predicted to read codon 3-A,G,C, and U by superwobbling [61,62,63]. Because valine occupies a 4-codon sector, such promiscuity would be tolerated and might be selected. The tRNA^Val^ (CAC) anticodon loop is substantially unwound by ValRS-IA, indicating allosteric effects of ValRS-IA-tRNA^Val^ binding. Allostery is likely important in selectively directing the tRNA^Val^ 3′-end to the aminoacylating or separate proofreading active site. Because ValRS-IA makes elbow contacts, these might leverage allosteric effects mediated through tRNA^Val^. It appears that 35-AC-36 and 38C make the strongest contact with ValRS-IA, as might be expected. In *P. furiosus*, 38A is present, rather than 38C, as in *E. coli*. In *P. furiosus*, tRNA^Val^ and tRNA^Ala^ are similar in sequence, consistent with the GADV hypothesis that indicates that valine and alanine were two of the first four encoded amino acids.

### 4.4. IleRS-IA

**Figure 7:**
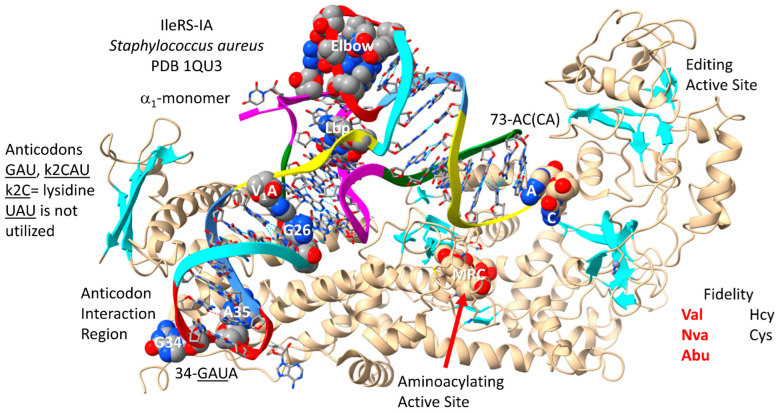
IleRS-IA-tRNA^Ile^ (GAU) from *S. aureus*. IleRS-IA has a separate proofreading active site that removes non-cognate homocysteine and cysteine attached to tRNA^Ile^ (black text). Non-cognate valine, norvaline and α-aminobutyrate are blocked from attachment to tRNA^Ile^ through reactions at the aminoacylating active site (red text) [36,37]. MRC binds the aminoacylating active site.

IleRS-IA-tRNA^Ile^ (GUA) from the Bacterium *Staphylococcus aureus* is shown in Figure 7 [71]. By structure and sequence, IleRS-IA is closely related to ValRS-IA. Both enzymes function as α_1_-monomers. Compared to ValRS-IA, IleRS-IA appears to make no elbow contacts and unwinds the tRNA^Ile^ (GAU and k2CAU (k2C stands for lysidine)) (in Archaea, GAU and agm2CAU (agm2C stands for agmatidine)) anticodon loop to a somewhat lesser extent than ValRS-IA. Elbow contacts by an AARS may be used to leverage allosteric effects transmitted through a cognate tRNA to the aminoacylating or editing active sites. Apparently, k2C and agm2C can partly mimic G for IleRS-IA binding. Minimally, k2C and agm2C are better G mimics than 2-C=O, as in unmodified C at the 2-carbon position (i.e., Met anticodons). The agm2C modification is added by a first protein (tRNA^Ile2^ 2-agmatinylcytidine synthase). As noted above, the UAU anticodon is rarely encoded in Archaea. When the anticodon UAU is encoded, it is also modified to agm2CAU to encode Ile. Also, MetRS-IA utilizes CmAU (elongator) and CAU (initiator) anticodons. Because Ile anticodons have tRNA-36U, tRNA-37 is t6A or hn6A. It is hypothesized that modified tRNA-37A (i.e., t6A and hn6A) may have evolved to suppress wobbling at tRNA-36U.

Because IleRS-IA is a class I AARS, the aminoacylating active site is at the C-terminal ends of a set of parallel β-sheets. The reaction intermediate analogue MRC binds at this site. 73-AC(CA) is not fully resolved in the structure. IleRS-IA has a separate editing active site that can remove non-cognate homocysteine and cysteine from tRNA^Ile^. The IleRS-IA aminoacylating active site limits incorporation of valine, norvaline and α-aminobutyric acid. Similar to valine, isoleucine is a somewhat featureless amino acid that requires editing functions to suppress translation errors.

**Figure 8:**
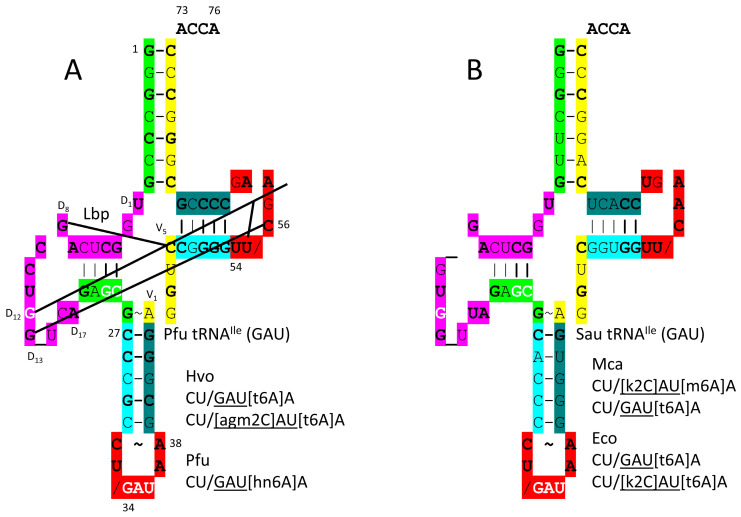
tRNA^Ile^ (GAU). (**A**) *P. furiosus* tRNA^Ile^ (GAU). (**B**) *S. aureus* tRNA^Ile^ (GAU). Modifications to the anticodon loop are as expected. Mca stands for *Mycoplasma capricolum*.

tRNA^Ile^ (GAU) is shown in Figure 8. In *P. furiosus*, tRNA^Ile^ (GAU) matches the primordial tRNA sequence in 4 bp within the acceptor stem. The D loop has the sequence D_1_-UGGCUCAGCCUGG_UCA-D_17_, matching the primordial tRNA in all but 6 positions, with a single base deleted from tRNA^Pri^. The 5′-As* sequence is GAGCG versus GGGCG in the primordial tRNA [15,31]. The anticodon stem matches the primordial tRNA in 3 bp. The T stem–loop–stem matches 4 of 5 bp in the stem and all but two bases in the loop compared to tRNA^Pri^. In *S. aureus*, the tRNA^Ile^ is more innovated.

### 4.5. MetRS-IA

**Figure 9:**
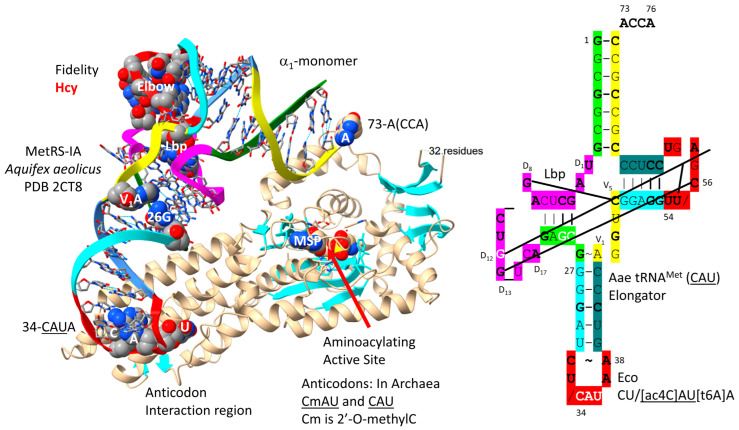
MetRS-IA-tRNA^Met^ (CAU) from *A. aeolicus*.

MetRS-IA-tRNA^Met^ (CAU) from *Aquifex aeolicus* is shown in Figure 9 [72]. In sequence and structure, MetRS-IA is very similar to IleRS-IA and ValRS-IA. MetRS-IA functions as an α_1_-monomer. There are no tRNA^Met^ (CAU) elbow contacts. Unwinding of the anticodon loop to expose CmAU or CAU is slight, perhaps because only a single CAU anticodon, lacking the agm2C or k2C modifications to encode isoleucine, is recognized. Because of deletion, MetRS-IA lacks an editing active site, but the aminoacylating active site of MetRS-IA limits incorporation of homocysteine. The aminoacylating active site is identified by MSP binding and a set of parallel β-sheets. 73-A(CCA) is partly resolved in the structure, possibly indicating allosteric effects (i.e., of MSP analogue binding).

It is hypothesized that methionine entered the genetic code by invading a 4-codon isoleucine sector [16]. This invasion resulted in the suppression of the UAU anticodon that would cause ambiguity between methionine and isoleucine coding. Methionine adopted the CmAU (elongator) and CAU (initiator) anticodons (Figure 8). In Archaea, to support a 3-codon box, isoleucine utilized the agm2CAU and GAU anticodons. In Bacteria, the k2CAU and GAU anticodons are utilized for isoleucine.

**Figure 10:**
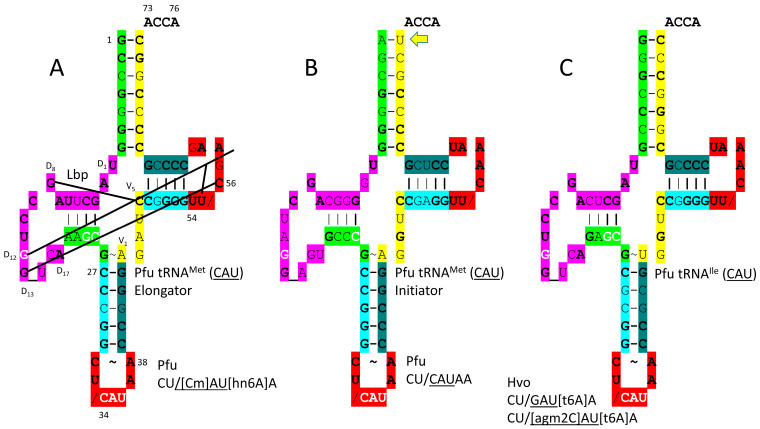
tRNA^Met^ (CmAU and CAU) and tRNA^Ile^ (agm2CAU). (**A**) *P. furiosus* elongator tRNA^Met^ (CmAU). (**B**) *P. furiosus* initiator tRNA^Met^ (CAU). (**C**) *P. furiosus* tRNA^Ile^ (agm2CAU).

The *P. furiosus* elongator tRNA^Met^ (CmAU) is a close match to the primordial tRNA sequence (Figure 10A). The acceptor stem matches in 5 bp out of 7. The D loop sequence D_1_-UAGCUUAGCCUGG_UCA-D_17_ matches in all but four positions, with a single base deleted from tRNA^Pri^. The anticodon stem matches tRNA^Pri^ in 4 out of 5 bp. The T stem matches in 4 of 5 bp, and the T loop matches tRNA^Pri^ in all but two bases. The initiator tRNA^Met^ (CAU) anticodon stem matches tRNA^Pri^ in 5 out of 5 bp (Figure 10B). The initiator tRNA^Met^ (CAU) anticodon loop is unmodified. The 1A-72U pair in the initiator tRNA^Met^ (CAU) is unusual (Figure 10B) and more readily melted than 1G=72C, which is typical. The tRNA^Ile^ (agm2CAU) sequence is similar to the elongator tRNA^Met^ (CmAU) (Figure 10C), indicating that tRNA^Met^ may have been derived from a primitive tRNA^Ile^.

### 4.6. LeuRS-IA

tRNA^Leu^ is a type II tRNA with a longer V arm (initially, 14 nt; a 3′-acceptor stem ligated to a 5′-acceptor stem; initially, CCGCCGC_GCGGCGG) (Figure 1). In Archaea, tRNA^Leu^ and tRNA^Ser^ are type II tRNAs. Both leucine and serine are in 6-codon boxes. LeuRS-IA and SerRS-IIA utilize the longer tRNA V arms as major determinants for cognate tRNA charging rather than the anticodon loops, which LeuRS-IA and SerRS-IIA do not contact. Arginine is also in a 6-codon box, but tRNA^Arg^ is a type I tRNA. Rather than using a longer type II tRNA^Arg^ V arm, ArgRS-IA uses enhanced anticodon loop unwinding to expose bases for recognition. In Bacteria, tRNA^Tyr^ is a type II tRNA, but the type II tRNA^Tyr^ (GUA) and its recognition by bacterial TyrRS-IC are bacterial innovations.

**Figure 11:**
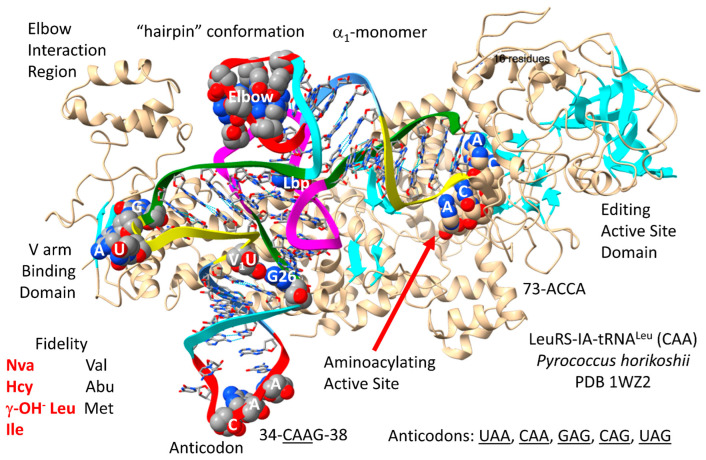
LeuRS-IA-tRNA^Leu^ (CAA) of *P. horikoshii*. LeuRS-IA has a separate editing active site that removes non-cognate valine, α-aminobutyric acid and methionine from tRNA^Leu^ (black text). The aminoacylating active site blocks norvaline, homocysteine, γ-hydroxy leucine and isoleucine incorporation (red text). In *P. horikoshii*, tRNA^Leu^ is a type II tRNA with a 14 nt V arm.

The LeuRS-IA-tRNA^Leu^ (CAA) of *Pyrococcus horikoshii* is shown in Figure 11 [73,74]. By structure and sequence, LeuRS-IA is closely related to ValRS-IA, IleRS-IA and MetRS-IA. LeuRS-IA functions as an α_1_-monomer. Archaeal and bacterial LeuRS-IA have different modes of contacting the type II V arm of their cognate tRNA^Leu^. In Archaea (but not in Bacteria), LeuRS-IA contacts the end loop of the V arm at the typical sequence UAG [11]. In both Archaea and Bacteria, the tRNA^Leu^ (CAA) anticodon loop is not contacted by LeuRS-IA. Of all the AARSs, only LeuRS-IA, SerRS-IIA and AlaRS-IID lack anticodon loop contacts for cognate tRNA recognition. A C-terminal region of archaeal LeuRS-IA contacts the elbow. The aminoacylating active site is identified by parallel β-sheets and 73-ACCA-76. The tRNA^Leu^ (CAA) 73-ACCA-76 is in the catalytic “hairpin” conformation for class I AARS enzymes, curving down into the aminoacylating active site. LeuRS-IA has a separate editing active site that removes non-cognate valine, α-aminobutyrate and methionine from tRNA^Leu^. The LeuRS-IA aminoacylating active site limits non-cognate norvaline, homocysteine, γ-OH^-^ leucine and isoleucine incorporation [36]. Leucine is a hydrophobic amino acid with little chemical character and so requires editing to maintain translational accuracy.

**Figure 12:**
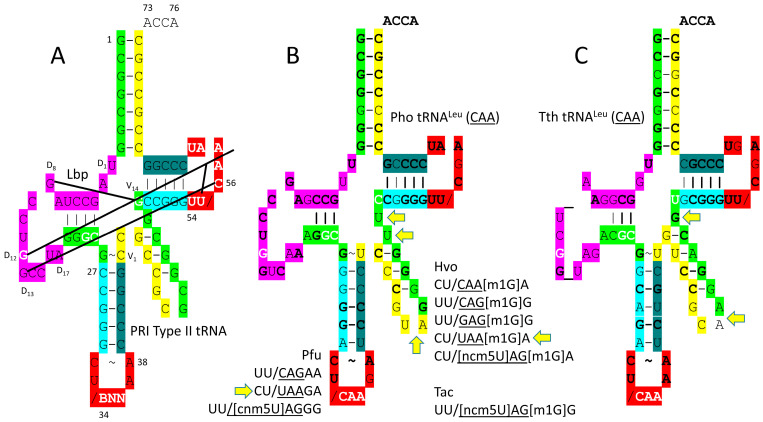
Comparison of type II tRNA^Pri^ and tRNA^Leu^. (**A**) Type II tRNA^Pri^. In the anticodon, B indicates G, C or U, but not A. (**B**) *P. horikoshii* tRNA^Leu^ (CAA). In Archaea, two bases separate the 3′-V arm stem from the Levitt base (V_14_C), giving the trajectory of the V arm. The V_6_-UAG-V_8_ consensus to bind LeuRS-IA is indicated. In principle, the unmodified CU/UAAGA anticodon loop could cause leucine substitution for phenylalanine by superwobbling. (**C**) Bacterial *T. thermophilus* tRNA^Leu^ (CAA) has a different trajectory of the V arm and lacks the V arm end loop UAG consensus. In Bacteria, the trajectory of the V arm is given by the number of unpaired bases (one) separating the 3′-V arm stem and the Levitt base V_15_U.

Figure 12 shows a comparison of a primordial type II tRNA (Figure 12A) and archaeal tRNA^Leu^ (CAA) (Figure 12B). A bacterial tRNA^Leu^ is also shown (Figure 12C). In *P. horikoshii*, the tRNA^Leu^ (CAA) type II V arm is 14 nt, as in type II tRNA^Pri^. Most archaeal tRNA^Leu^ type II V arms are 14 nt in length, the primordial length. In Archaea, the tRNA^Leu^ type II V arm is a major determinant for cognate tRNA^Leu^ charging with leucine. The trajectory of the type II V arm is different than that for tRNA^Ser^, with two bases separating the 3′-V arm stem and the Levitt base for tRNA^Leu^ and with one base separating the 3′-V arm stem and the Levitt base for tRNA^Ser^, in Archaea. For archaeal tRNA^Leu^, the V arm end loop includes the UAG consensus to bind LeuRS-IA (Figure 12B). The V arm end loop contact is not utilized by bacterial LeuRS-IA (Figure 12C). tRNA^Leu^ utilizes CAA, UAA, CAG, GAG and UAG anticodons. Because of superwobbling, unmodified UAA anticodons, in principle, might utilize a phenylalanine UUU or UUC codon, substituting leucine for phenylalanine. We are not certain what limits miscoding in this case. The anticodon cnm5UAG is in a 4-codon box, but it has the 5-carbon U modification that should suppress superwobbling.

The *P. horikoshii* tRNA^Leu^ (CAA) acceptor stem matches tRNA^Pri^ in 6 out of 7 bp (Figure 12B). The D loop has the sequence D_1_-UUGCCGAGCCUGGUCAA-D_17_, matching the tRNA^Pri^ sequence in all but four positions and including no deletions compared to tRNA^Pri^. The 5′-As* sequence is AGGCG, matching the typical tRNA^Pri^ GGGCG in all but one position. Two bases separate the type II 3′-V arm stem and the Levitt base. The V arm end loop has the sequence V_5_-GUAG-V_8_ that includes the V_6_-UAG-V_8_ consensus to bind LeuRS-IA (Figure 11 and Figure 12B). The T stem matches tRNA^Pri^ all but 1 bp. The T loop matches in all but one base.

### 4.7. SerRS-IIA

At the base of code evolution, only tRNA^Leu^ and tRNA^Ser^ were selected to be type II tRNAs. The number of amino acids that are type II in an organism or domain is determined by the allowed trajectories of the V arm. In Archaea, the number of trajectories is two. In Bacteria, the number is three. In Archaea and Bacteria, the trajectories of type II V arms are different for tRNA^Leu^ and tRNA^Ser^. SerRS-IIA is a very different AARS compared to LeuRS-IA. From sequences, however, it appears that type II tRNA^Ser^ may have been derived from type II tRNA^Leu^. To maintain translational accuracy, the type II V arm of tRNA^Ser^ is recognized very differently than the type II V arm of tRNA^Leu^. The type II tRNA^Ser^ V arm has a different trajectory from its tRNA body compared to the tRNA^Leu^ V arm [11]. The trajectory of the type II V arm depends on the number of unpaired bases between the 3′-V arm stem and the Levitt reverse Watson–Crick base pair (i.e., D_8_G=V_14_C). In Archaea, for tRNA^Leu^, the number is two unpaired bases (in Bacteria, the number is one unpaired base for the tRNA^Leu^ type II V arm). For tRNA^Ser^, the number in Archaea is one unpaired base (in Bacteria, the number is zero unpaired bases for the tRNA^Ser^ type II V arm).

**Figure 13:**
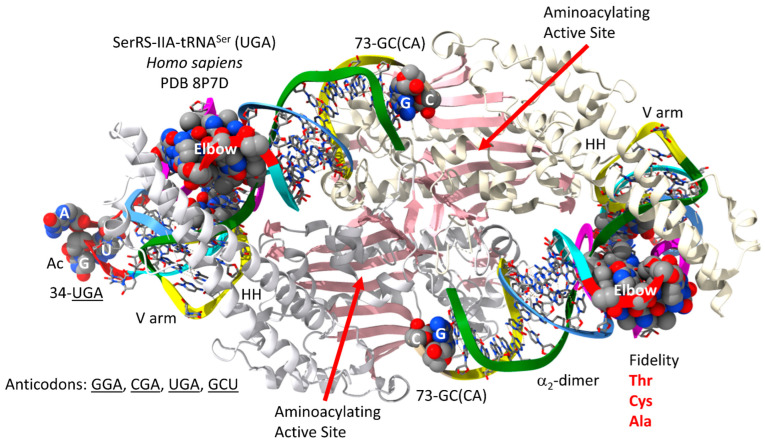
SerRS-IIA-tRNA^Ser^ (UGA) from *H. sapiens*. The full α_2_-dimer is shown. One α-subunit is colored white; one is colored wheat. β-sheets are colored light pink. HH indicates the N-terminal helix hairpin that binds the type II V arm stems and the elbow of tRNA^Ser^.

The human SerRS-IIA-tRNA^Ser^ (UGA) is shown in Figure 13 [75]. SerRS-IIA has an N-terminal helix hairpin that lies across the type II V arm and interacts with the tRNA^Ser^ elbow. SerRS-IIA functions as an α_2_-dimer. The aminoacylating active site and the helix hairpin for a single tRNA^Ser^ are on separate α-subunits. As noted above, no contact is made by SerRS-IIA with the tRNA^Ser^ anticodon loop. Instead, SerRS-IIA recognizes the type II V arm as a major determinant. The aminoacylating active site is on the surface of antiparallel β-sheets. The SerRS-IIA aminoacylating active site limits non-cognate attachment of threonine, cysteine and alanine to tRNA^Ser^.

**Figure 14:**
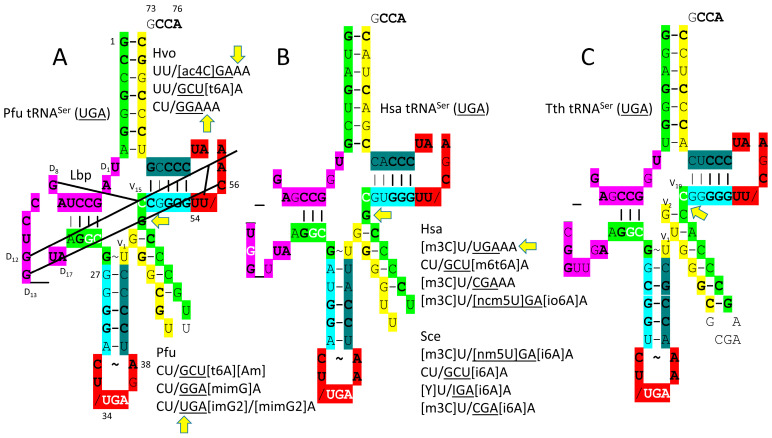
tRNA^Ser^ (UGA). (**A**) *P. furiosus* tRNA^Ser^ (UGA). In Archaea, one base separates the 3′-V stem and the Levitt base (in this case, V_15_C). (**B**) *H. sapiens* tRNA^Ser^ (UGA). (**C**) *T. thermophilus* tRNA^Ser^ (UGA). In Bacteria, typically, zero bases separate the 3′-V arm stem and the Levitt base (V_19_C).

tRNA^Ser^ anticodon loop modifications are interesting and slightly unanticipated (Figure 14). Generally, tRNA-36U is associated with a modified tRNA-37A, as is observed. Generally, tRNA-36A is associated with a modified tRNA-37G, but GGA in *H. volcanii* is followed by unmodified 37A. In *P. furiosus*, UGA is unmodified at 34U, but UGA is in a 4-codon box, so implied superwobbling would not cause miscoding. In the *P. furiosus* tRNA^Ser^ (UGA), the acceptor stem matches that of tRNA^Pri^ in 4 of 7 bp. In *H. sapiens*, tRNA^Ser^ (UGA) matches the tRNA^Pri^ acceptor stem in 5 of 7 bp (Figure 14B). In the D loop, tRNA^Ser^ (UGA) of *P. furiosus* has two perfect UAGCC repeats, consistent with the three 31 nt tRNA evolution theorem (Figure 1 and Figure 2). The D loop sequence is D_1_-UAGCCUAGCCUGG__UA-D_17_, matching the primordial tRNA sequence in all but two deleted positions. The 5′-As* sequence AGGCG matches tRNA^Pri^ in 4 of 5 positions. The T stem–loop–stem of *P. furiosus* matches tRNA^Pri^ in all but 1 stem bp.

In Bacteria, trajectories of tRNA type II V arms are different than in Archaea (compare Figure 14A,C). In Archaea, tRNA^Leu^ has two unpaired bases (Figure 12B), and tRNA^Ser^ has one unpaired base (Figure 14A) separating the 3′-V arm stem and the Levitt base. In Bacteria, tRNA^Tyr^ has two unpaired bases (see below), tRNA^Leu^ has one unpaired base (Figure 12C), and tRNA^Ser^ has zero unpaired bases (Figure 14C) separating the type II V arm 3′-stem and the Levitt base [11]. Differences in type II V arm trajectories cause different modes of cognate AARS-tRNA recognition and are expected to limit horizontal type II tRNA gene transfers between Bacteria and Archaea.

Serine is the only amino acid that is located at two separate columns of the genetic code. It is hypothesized that serine jumped from column 2 to column 4 during code evolution. Being a type II tRNA lacking anticodon recognition by SerRS-IIA probably facilitated jumping. We suggest that jumping of serine in code evolution may correlate with the introduction of cysteine into the code (see below).

### 4.8. ArgRS-IA

**Figure 15:**
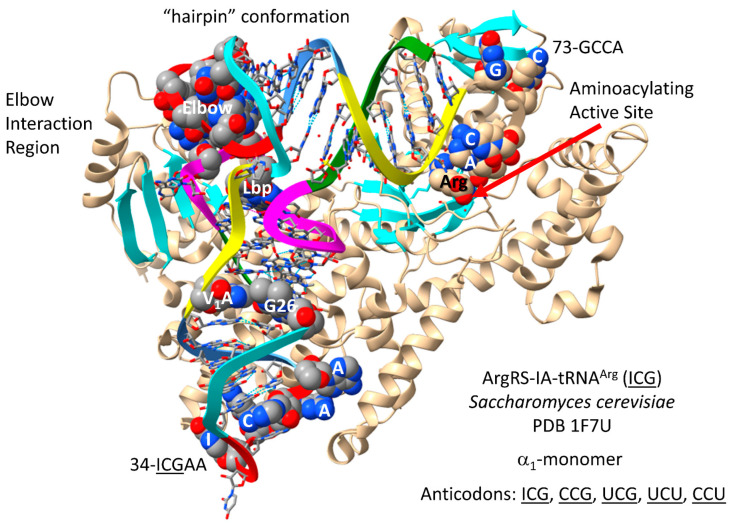
ArgRS-IA-tRNA^Arg^ (ICG) of *S. cerevisiae*.

The ArgRS-IA-tRNA^Arg^ (ICG) of *S. cerevisiae* is shown in Figure 15 [76]. As noted above, although arginine is in a 6-codon box, tRNA^Arg^ is a type I tRNA. Compared to LeuRS-IA and SerRS-IIA, ArgRS-IA utilizes the alternate strategy of increased unwinding of the type I tRNA^Arg^ anticodon loop to expose additional bases for cognate recognition. Three amino acids probably could not occupy 6-codon sectors in the code using the strategy that evolved for arginine, explaining why tRNA^Leu^ and tRNA^Ser^ evolved to substitute recognition of longer type II V arms, rather than utilizing anticodon loop determinant contacts. For tRNA^Arg^, the 34-ICGAA-38 sequence is substantially unwound. 35C, 37A and 38A appear to make strong contacts with ArgRS-IA. ArgRS-IA makes substantial elbow contacts that may help leverage anticodon loop opening through allosteric effects. 73-GCCA-76 is in the catalytic “hairpin” conformation for a class I AARS. Arginine binds at the aminoacylating active site. As expected for a class I AARS, parallel β-sheets approach the aminoacylating active site.

In *P. furiosus*, the GCG anticodon would most closely correspond with the ICG anticodon in *S. cerevisiae*. Generally, when encoded ACG is modified to ICG by deamination, in Bacteria and Eukarya, the corresponding GCG anticodon is not utilized. Similarly, Archaea do not appear to utilize the 34A→I modification but use the GCG anticodon instead. It is notable that 34A is not utilized in Archaea and, for the most part, in Bacteria (Bacteria utilize ICG to encode arginine). The lack of anticodon wobble base discrimination (i.e., pyrimidine versus purine only) causes genetic code degeneracy.

**Figure 16:**
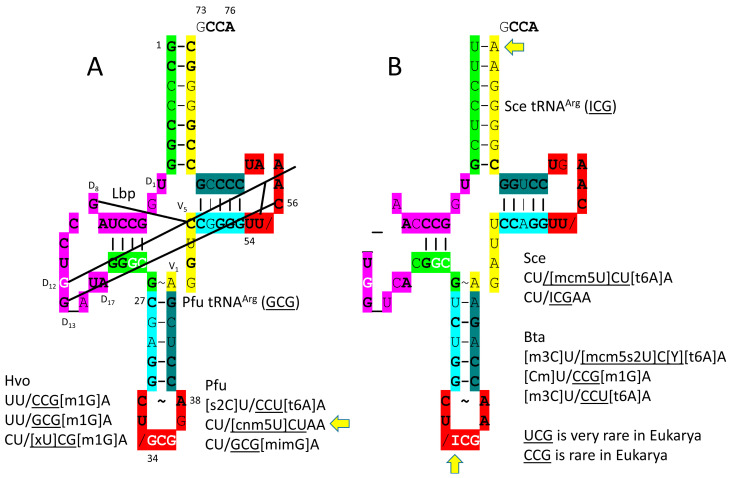
tRNA^Arg^. (**A**) *P. furiosus* tRNA^Arg^ (GCG). (**B**) *S. cerevisiae* tRNA^Arg^ (ICG). Yellow arrows indicate features of possible interest. Bta indicates *Bos taurus*.

The *P. furiosus* tRNA^Arg^ (GCG) sequence is of interest (Figure 16A). The acceptor stem matches tRNA^Pri^ in 5 of 7 bp. The D loop has the sequence D_1_-UGGCCUAGCCUGG_AUA-D_17_, which varies in only three positions from the primordial D loop sequence, with a single base deleted relative to tRNA^Pri^. The 5′-As* sequence is GGGCG, which matches the primordial tRNA sequence (GGGCG, rearranged from GGCGG before LUCA). The V loop sequence AGGUC is typical. The T stem–loop–stem matches in all but 1 stem bp. The *S. cerevisiae* tRNA^Arg^ (ICG) sequence is more derived from the root sequence, as expected (Figure 16B). In *P. furiosus*, the [cnm5U]CU anticodon is followed by an unmodified A, which is unexpected. Perhaps the cnm5U modification, or another feature, helps to compensate. Generally, 36U is followed by a modified 37A, as in CCU[t6A]. CGA and CGG codons are rare in Eukarya. The corresponding UCG and CCG anticodons are also rare in Eukarya.

Arginine is an amino acid with significantly discriminating characteristics. Arginine is positively charged and much stiffer than lysine. Also, arginine has significant hydrogen bonding potential. These characteristics discriminate arginine from lysine, which is much more flexible and has a more concentrated positive charge. We consider the idea that the first encoded positively charged amino acid may have been ornithine [77]. Ornithine can be converted to arginine in two enzymatic steps, consistent with the notion that tRNA-linked chemistry may have contributed to the encoding of arginine and lysine. Ornithine can be converted to lysine in some Archaea and Bacteria [78,79,80]. Consistent with this idea, in *P. furiosus*, tRNA^Arg^ and tRNA^Lys^ are similar in sequence.

### 4.9. CysRS-IA

**Figure 17:**
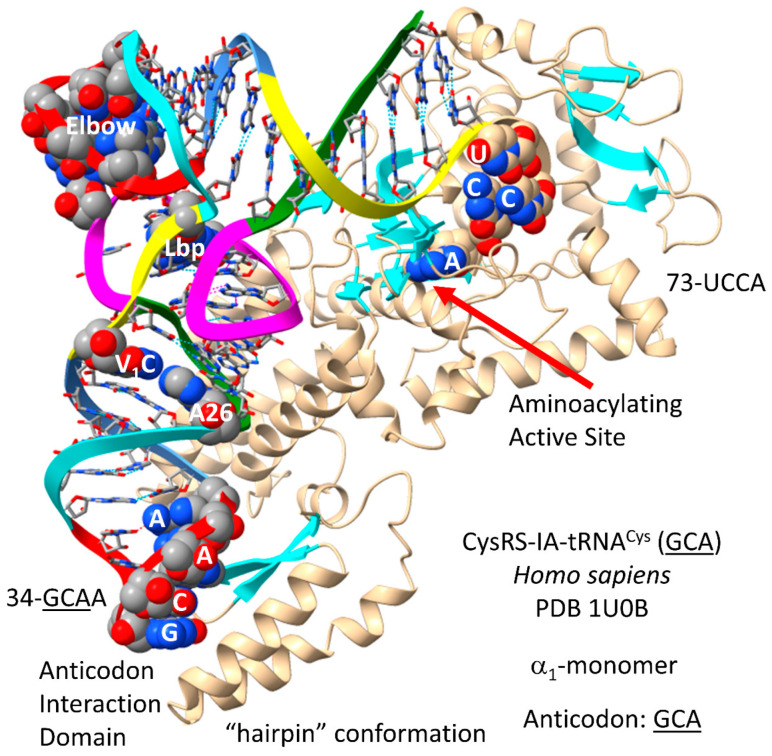
CysRS-IA-tRNA^Cys^ (GCA) from *H. sapiens*.

In sequence and structure, CysRS-IA (Figure 17) is closely related to ArgRS-IA. Cysteine and arginine are located at column 4 of the genetic code, indicating evolution in code columns. Because CysRS-IA recognizes only the GCA anticodon, 34-GCA-36 can be recognized by the anticodon-binding domain [81]. In the structure, 73-UCCA-76 enters the CysRS-IA aminoacylating active site in the “hairpin” catalytic conformation. The discriminator 73U is rarely used. In *P. furiosus*, 73U is only found in tRNA^Cys^ (1 tRNA^Cys^) and tRNA^Thr^ (3 tRNA^Thr^). The aminoacylating active site of CysRS-IA is at the C-terminal ends of a set of parallel β-sheets, as expected. Cysteine is important for Zn binding. CysRS-IA utilizes Zn binding to bind and orient cysteine in its aminoacylating active site.

**Figure 18:**
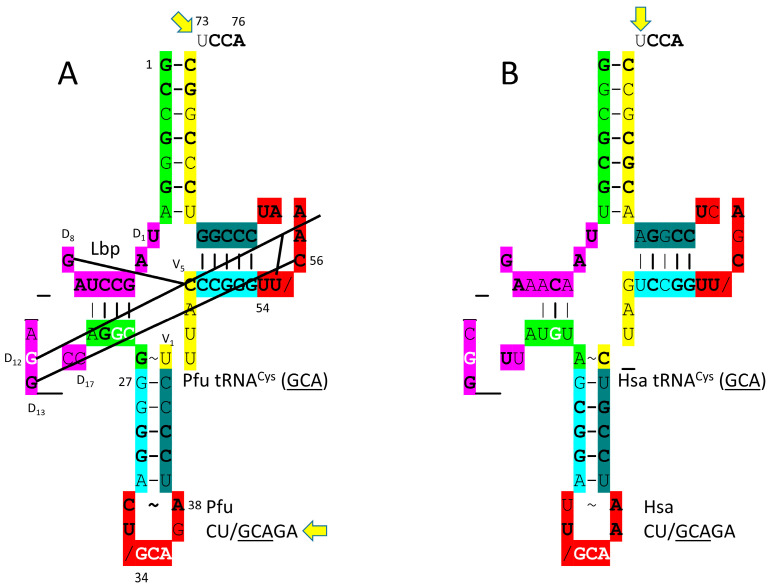
tRNA^Cys^ (GCA). (**A**) *P. furiosus* tRNA^Cys^ (GCA). (**B**) *H. sapiens* tRNA^Cys^ (GCA).

In *P. furiosus*, tRNA^Cys^ (GCA) is of interest (Figure 18). The acceptor stem matches tRNA^Pri^ in 4 of 7 bp. The D loop sequence is D_1_-UAGCCUAG__AGG__CC-D_17_, matching the primordial tRNA sequence in the first eight positions. The 5′-As* sequence is AGGCG, matching tRNA^Pri^ GGGCG in 4 of 5 positions. The anticodon stem matches tRNA^Pri^ in 2 bp. The T stem–loop–stem matches the primordial tRNA sequence exactly. Interestingly, for 34-GCAG, the anticipated modified 37G is not present. The *P. furiosus* tRNA^Cys^ (GCA) is very similar to the human tRNA^Cys^ (GCA), possibly indicating a monophyletic relationship between tRNA^Cys^ in Archaea and Eukarya.

Cysteine may have first entered the genetic code by tRNA-linked chemistry. There are two mechanisms by which Ser-tRNA^Cys^ might be converted to Cys-tRNA^Cys^. pSer-tRNA^Cys^ can be converted to Cys-tRNA^Cys^ by pSer-tRNA^Cys^→Cys-tRNA^Cys^ cysteine synthase (pSer stands for o-phosphoserine) [82]. Serine can also be acetylated and then converted to cysteine with H_2_S. It is hypothesized that serine jumping to column 4 of the genetic code from column 2 may have resulted from such a tRNA-linked mechanism. Cysteine ended up in column 4, row 1. Most row 1 amino acids (i.e., Phe, Tyr, Trp and Cys) appear to be among the last encoded. Cysteine, however, was important for Zn binding and protein folding (i.e., for AARS enzymes), indicating that cysteine must have entered the code earlier, before landing in its row 1 location [15,16,17]. Serine may have occupied a larger sector of column 2 (i.e., rows 2 and 3). Serine or serine converted to cysteine may have jumped to row 4 (i.e., from column 2, row 3A (GGU) to column 4, row 3A (GCU)). Serine converted to cysteine could have shifted to column 4, row 1 (GCU→GCA), and CysRS-IA could have evolved from a primitive ArgRS-IA. In this manner, cysteine could have entered the code early with tRNA-linked synthesis but found its eventual position late. The GCU within a disrupted arginine sector would then have reverted to a serine anticodon. In column 2 of the code, Thr and Pro displaced Ser to its location in column 2, row 1. SerRS-IIA recognizes a type II tRNA^Ser^, without anticodon recognition. A simple change in the tRNA^Ser^ anticodon might, therefore, be sufficient to achieve the jump from column 2 to column 4, but the change in the anticodon would not affect SerRS-IIA recognition. Serine split what was probably an enlarged arginine sector by jumping into column 4. The jumping of serine from column 2 to column 4 was some of the only chaos in generating the standard code.

### 4.10. ThrRS-IIA

**Figure 19:**
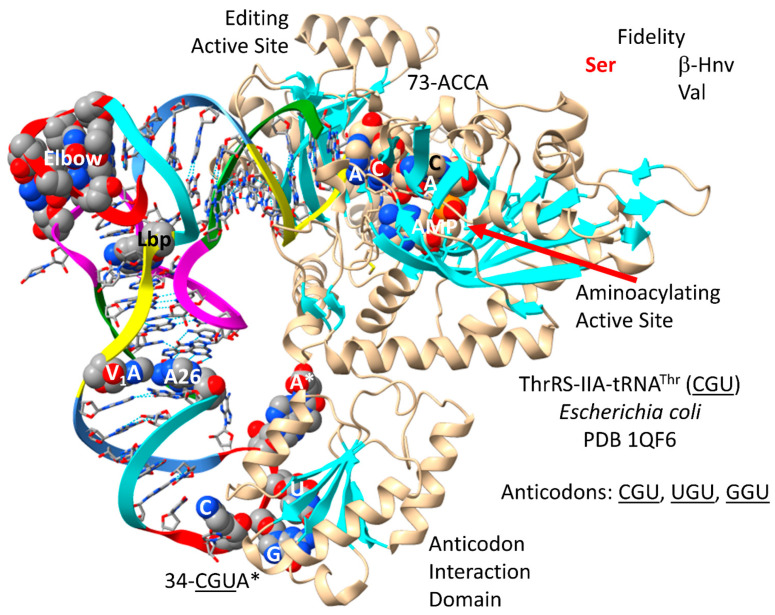
The ThrRS-IIA-tRNA^Thr^ (CGU) from *E. coli*. A* is 37m6t6A.

By structure and sequence, ThrRS-IIA (Figure 19) is very similar to SerRS-IIA and GlyRS-IIA. As a class II AARS, ThrRS-IIA has its aminoacylating active site on a surface of antiparallel β-sheets [83]. 73-ACCA-76 penetrates the aminoacylating active site, where AMP binds. In *P. furiosus*, the discriminator base is 73U rather than 73A, as in *E. coli*. ThrRS-IIA has a separate editing active site that removes non-cognate β-hydroxynorvaline and valine from tRNA^Thr^. The aminoacylating active site of ThrRS-IIA limits non-cognate attachment of serine to tRNA^Thr^. The anticodon binding region of ThrRS-IIA binds 35-GU[m6t6A]-37 (in *E. coli*).

**Figure 20:**
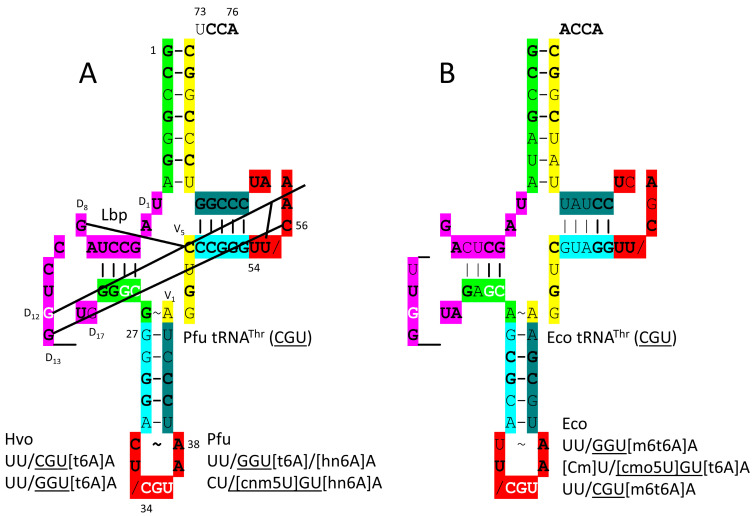
tRNA^Thr^ (CGU). (**A**) *P. furiosus* tRNA^Thr^ (CGU). (**B**) *E. coli* tRNA^Thr^ (CGU).

tRNA^Thr^ (CGU) is shown in Figure 20. In *P. furiosus*, the acceptor stem matches the primordial tRNA sequence in 4 of 7 bp. The D loop has the sequence UAGCCUAGCCUGG__UG, which matches the primordial tRNA sequence in the first 13 positions exactly and in all but three positions, two of which are deletions. The 5′-As* sequence is GGGCG, which is typical. The anticodon stem matches tRNA^Pri^ in 2 bp. The V loop sequence AGGUC is typical. In *P. furiosus*, the T stem–loop–stem matches the primordial tRNA sequence exactly. In Archaea and Bacteria, 36U is generally associated with a modified 37A (i.e., t6A or hn6A), as is observed. The modification of 37A may aid in accurate tRNA^Thr^ charging. Also, the modification of 37A may help to support the reading of 36U anticodons. In *P. furiosus*, tRNA^Thr^ resembles tRNA^Ser^ in sequence, except for the V loop region (tRNA^Thr^ is type I; tRNA^Ser^ is type II) [21].

### 4.11. ProRS-IIA

**Figure 21:**
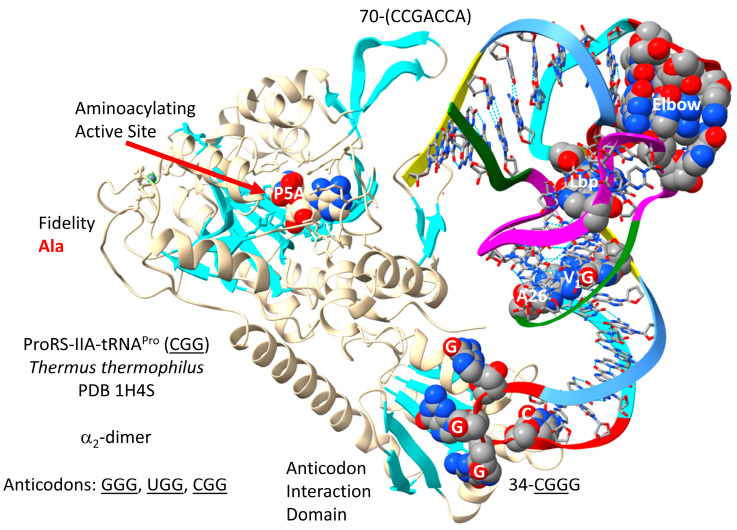
ProRS-IIA-tRNA^Pro^ (CGG) from *T. thermophilus*. P5A is a reaction intermediate analogue that binds in the aminoacylating active site.

ProRS-IIA-tRNA^Pro^ (CGG) is shown in Figure 21 [84]. ProRS-IIA is closely related to GlyRS-IIA, SerRS-IIA and ThrRS-IIA in sequence and structure. The aminoacylating active site is on the surface of antiparallel β-sheets. The reaction intermediate analogue P5A is located at the aminoacylating active site. In the structure, 70-(CCGACCA)-76 is disordered. The anticodon loop 34-CGGG-37 is substantially unwound, indicating allosteric effects, which may also be indicated by a disorder of the tRNA 3′-end. As expected, anticodon loop 35-GGG-37 makes the strongest ProRS-IIA binding contacts.

*T. thermophilus* and *P. furiosus* ProRS-IIA lack a separate editing active site that is, however, present in more derived Bacteria, such as *E. coli*. The aminoacylating active site of ProRS-IIA limits non-cognate alanine attachment to tRNA^Pro^.

**Figure 22:**
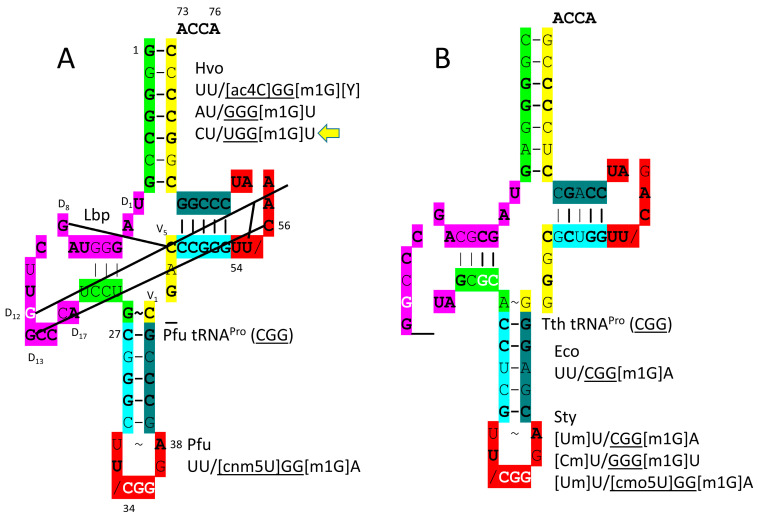
tRNA^Pro^ (CGG). (**A**) *P. furiosus* tRNA^Pro^ (CGG). (**B**) *T. thermophilus* tRNA^Pro^ (CGG). Sty indicates *Salmonella typhimurium*.

The *P. furiosus* tRNA^Pro^ (CGG) matches the acceptor stem of the primordial tRNA in 5 of 7 positions (Figure 22). tRNA^Pro^ (CGG) has the D loop sequence D_1_-UAGGGUAGCUUGGCCCA-D_17_, which matches the primordial D loop in all but four positions and has no deleted bases relative to tRNA^Pri^. The anticodon stem matches the primordial sequence in 3 of 5 bp. The V loop sequence C_GAC matches the primordial sequence CCGCC in three positions. The T stem–loop–stem sequence matches the primordial tRNA sequence exactly. Proline is in a 4-codon box and so utilizes CGG, GGG and UGG anticodons. Modifications are as expected, except that *H. volcanii* 34U is unmodified. Because proline occupies a 4-codon box, superwobbling need not necessarily be suppressed. By contrast, *P. furiosus* has the 34cnm5U modification to suppress superwobbling.

### 4.12. AspRS-IIB

**Figure 23:**
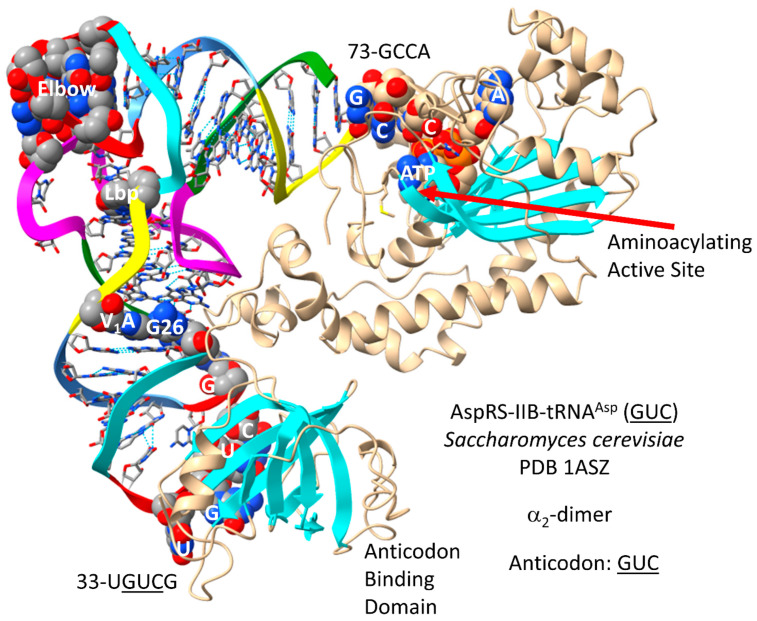
AspRS-IIB-tRNA^Asp^ (GUC) from *S. cerevisiae*.

A primitive AspRS may be the founding AARS in column 3 of the code (tRNA-35U). The AspRS-IIB-tRNA^Asp^ (GUC) of *S. cerevisiae* is shown in Figure 23 [85]. Column 3 is the most innovated column, dividing into 2-codon sectors. For tRNA^Asp^, only the GUC anticodon is utilized. The anticodon loop is substantially unwound, exposing 33-UGUCG-38 to make AspRS-IIB contacts. Anticodon loop unwinding indicates allosteric effects communicated to the AspRS-IIB aminoacylating active site through tRNA^Asp^ (GUC). 73-GCCA-76 enters the aminoacylating active site, where ATP binds. As expected, a surface of antiparallel β-sheets is present at the aminoacylating active site.

**Figure 24:**
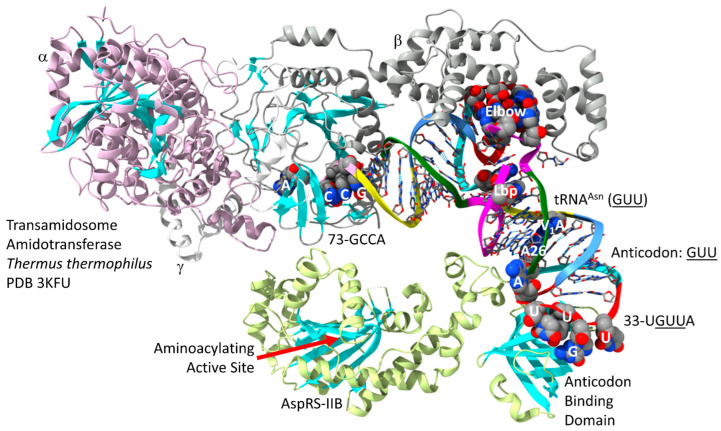
tRNA-linked chemistry. A detail of the *T. thermophilus* transamidosome is shown.

In Figure 24, part of a *T. thermophilus* transamidosome is shown [86]. This image provides a partial approximation of the mechanism by which asparagine and glutamine may have first entered the genetic code [87]. The α-subunit of the amidotransferase that modifies Asp-tRNA^Asn^ to Asn-tRNA^Asn^ (GUU) is homologous to an archaeal amidotransferase. Both asparagine and glutamine initially entered the code by tRNA-linked amidotransferase reactions. The tRNA^Asn^ (GUU) anticodon loop is substantially unwound. 33-UGUUA-37 interacts with the AspRS-IIB anticodon-binding domain.

Asp and Glu are closely related negatively charged amino acids that are located at column 3, row 4. Asp has a shorter side chain than Glu and so generally forms better ion pair allosteric switches, particularly with Arg, which is stiffer than Lys. In *P. furiosus*, tRNA^Asp^, tRNA^Glu^ and tRNA^Gln^ are all closely related tRNAs by sequence [21]. In *P. furiosus*, tRNA^Asn^ is most similar to tRNA^Tyr^. The deviation of tRNA^Asn^ from tRNA^Asp^ supports the discrimination of chemically similar amino acids in coding.

**Figure 25:**
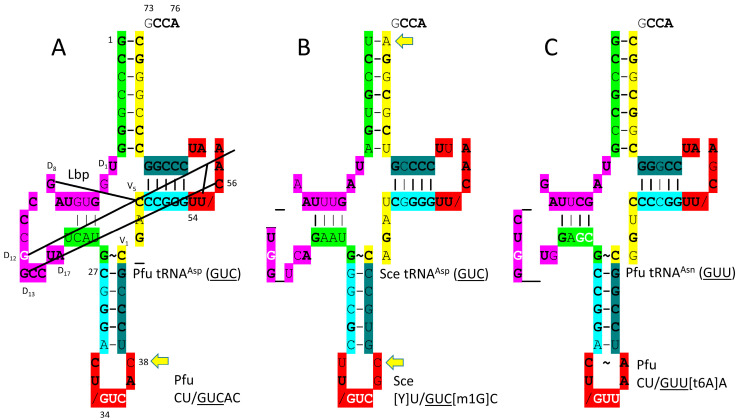
tRNA^Asp^ (GUC) and tRNA^Asn^ (GUU). (**A**) *P. furiosus* tRNA^Asp^ (GUC). (**B**) *S. cerevisiae* tRNA^Asp^ (GUC). (**C**) *P. furiosus* tRNA^Asn^ (GUU).

In Figure 25, tRNA^Asp^ (GUC) and tRNA^Asn^ (GUU) are compared. It is hypothesized that tRNA^Asn^ (GUU) evolved from tRNA^Asp^ (GUC) and that AsnRS-IIB evolved from AspRS-IIB by duplication and divergence. The acceptor stem of the *P. furiosus* tRNA^Asp^ (GUC) matches the primordial sequence in 4 of 7 bp. The D loop has the sequence D_1_-UGGUGUAGCCCGGCCUA-D_17_, which differs in four positions from the primordial tRNA but includes no deletions relative to tRNA^Pri^. The D loop sequence D_6_-UAGCCCGGCCUA-D_17_ has only a single mismatch compared with tRNA^Pri^. The anticodon stem matches tRNA^Pri^ in 3 bp. The T stem–loop–stem exactly matches the primordial tRNA sequence. The tRNA^Asp^ (GUC) anticodon loop has a 32C-38C arrangement, which should alter the dynamics of the loop relative to 32C-38A, which is most common and primordial. The *P. furiosus* tRNA^Asn^ (GUU) matches the acceptor stem of the primordial tRNA in 5 of 7 bp. The D loop has the sequence D_1_-UAGCUUAG_CUGG__UG-D_17_, with three bases deleted from the primordial tRNA D loop but matching in sequence in all but five positions. The 5′-As* sequence GAGCG matches the primordial sequence GGGCG in all but one base. The anticodon stem matches the primordial tRNA in 4 of 5 bp. The V loop sequence CGGUC matches tRNA^Pri^ CCGCC in 3 of 5 positions. The T stem–loop–stem matches in all but 1 stem bp and one loop base.

### 4.13. HisRS-IIA

**Figure 26:**
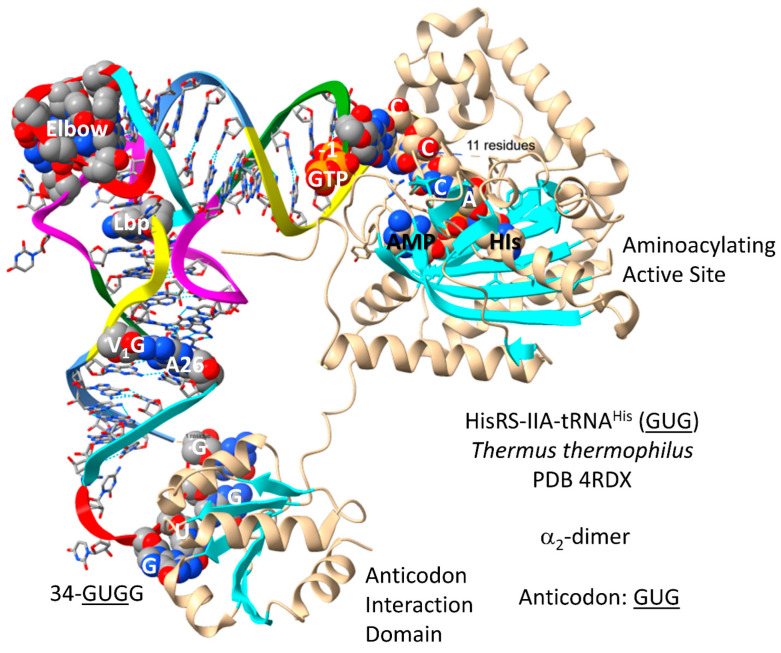
HisRS-IIA-tRNA^His^ (GUG) from *T. thermophilus*.

Another column-3 amino acid is histidine. HisRS-IIA-tRNA^His^ (GUG) from *T. thermophilus* is shown in Figure 26 [88]. HisRS-IIA functions as an α_2_-dimer. As a class II AARS, HisRS-IIA has the aminoacylating active site on the surface of antiparallel β-sheets. AMP and histidine bind in the aminoacylating active site, and 73-CCCA-76 enters the aminoacylating active site. On the ribosome, 74-CC-75 must pair with a GG sequence in the peptidyl site (P-site) of the peptidyl transferase center to orient the peptide–tRNA. Having the sequence 73-CCCA-76 in a tRNA, therefore, might cause problems with orienting the growing peptide chain during translation. To block 73C pairing with the ribosome G, tRNA^His^ (GUG) is modified by the addition of GTP at the -1 position. The enzyme that catalyzes this reaction is tRNA^His^ (-1) GTP transferase. This enzyme appears to be a first protein, as old as the genetic code. Also, the 73C=(-1)GTP base pair is a unique discriminator for the cognate tRNA^His^ (GUG) charging with histidine. As is also observed for tRNA^Asp^ (GUC) and tRNA^Asn^ (GUU), the tRNA^His^ (GUG) anticodon loop is unwound, exposing 34-GUGG-37 to bind the HisRS-IIA anticodon-binding domain. It is hypothesized that AspRS-IIB was originally AspRS-IIA but diverged to suppress tRNA charging errors. In this case, HisRS-IIA would have been derived from an AspRS-IIA prior to divergence.

**Figure 27:**
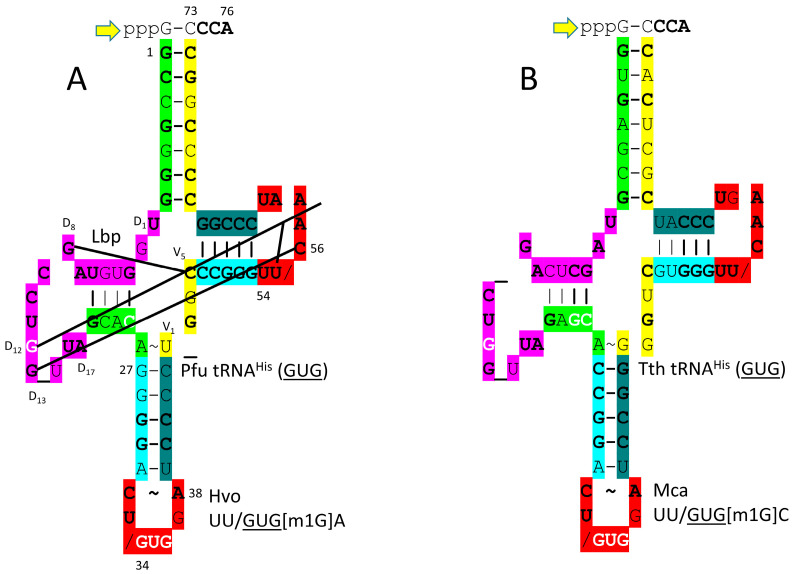
tRNA^His^ (GUG). (**A**) *P. furiosus* tRNA^His^ (GUG). (**B**) *T. thermophilus* tRNA^His^ (GUG). The yellow arrows indicate the unique (-1)GTP=73C discriminators that also may suppress misalignment of the P-site peptide–tRNA on the ribosome.

In *P. furiosus*, the tRNA^His^ (GUG) acceptor stem differs in only 2 bp from the primordial tRNA sequence (Figure 27). The D loop of tRNA^His^ (GUG) has the sequence D_1_-UGGUGUAGCCUGG_UUA-D_17_, differing in five positions from the primordial tRNA sequence, with a single base deletion relative to tRNA^Pri^. In *P. furiosus*, the anticodon stem matches tRNA^Pri^ in 2 bp. In *T. thermophilus*, the anticodon stem matches tRNA^Pri^ in 4 bp. In *P. furiosus*, the T stem–loop–stem exactly matches the primordial tRNA sequence.

### 4.14. GluRS-IB

Column 3 of the genetic code is the most innovated column that encodes the most amino acids. It appears that column 3 may have been sectored into 2-codon boxes initially by splitting Asp and Glu into a striped pattern of Asp in A rows (row 2A, 3A and 4A) and Glu in B rows (row 2B, 3B and 4B). A and B rows represent wobble tRNA-34. tRNA-34G is the anticodon base of the A row. At the base of code evolution, wobble tRNA-34A is rarely or never used. tRNA-34C or 34U is the anticodon base of the B row. Note that related amino acids and AARS enzymes Asp, Asn and His, charged to their cognate tRNAs by related enzymes AspRS-IIB, AsnRS-IIB and HisRS-IIA, are located at rows 4A, 3A and 2A. It is likely that AspRS was initially AspRS-IIA, which evolved to AspRS-IIB to suppress translation errors. Glu, Lys and Gln are located at rows 4B, 3B and 2B. GluRS-IB and LysRS-IB (in Archaea) are closely related enzymes. GlnRS-IB was derived from GluRS-IB in Eukarya (~2.5 billion years ago) and then transferred to many prokaryotic species by horizontal gene transfers. At LUCA, GluRS-IB added glutamate to tRNA^Gln^. Glu-tRNA^Gln^ was converted to Gln-tRNA^Gln^ by an amidotransferase. This is a similar tRNA-linked chemistry mechanism to that by which asparagine first entered the code [87,89,90,91].

**Figure 28:**
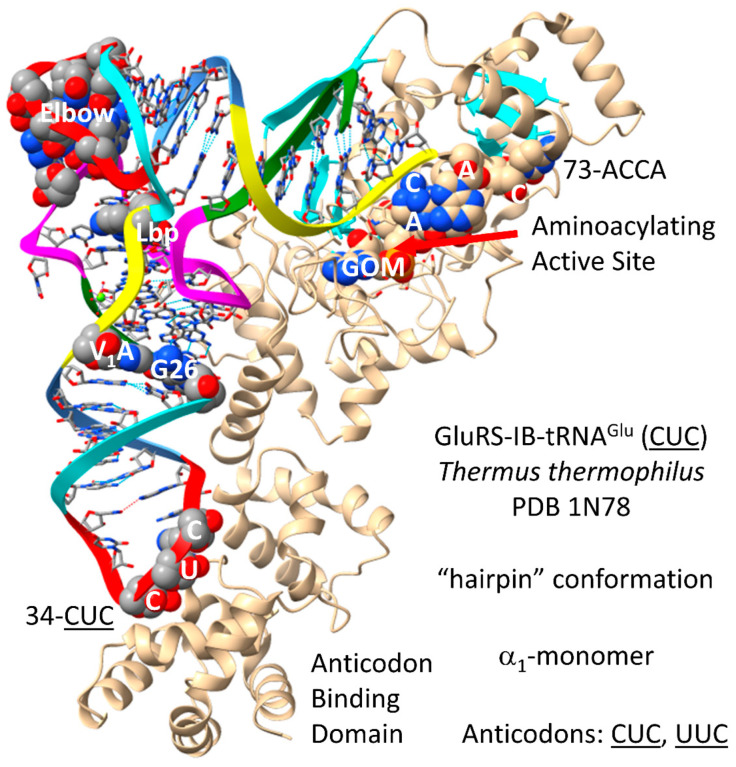
GluRS-IB-tRNAGlu (CUC) from *T. thermophilus*.

GluRS-IB (Figure 28) [92] may be derived from a primitive ArgRS-IA by duplication and repurposing. In contrast to AspRS-IIB, which is a class II AARS and an α_2_-dimer, GluRS-IB is a typical class I AARS that functions as an α_1_-monomer. The GluRS-IB aminoacylating active site is at the C-terminal ends of a set of parallel β-sheets. 73-ACCA-76 penetrates to the aminoacylating active site in the catalytic hairpin conformation for class I AARSs. The non-reactive GOM synthetic reaction intermediate binds here. In contrast to AspRS-IIB and AsnRS-IIB, the anticodon loop of tRNA^Glu^ is not substantially unwound by GluRS-IB. This difference and the difference of discriminator bases (73G (i.e., Asp) versus 73A (i.e., Glu)) may contribute to Asp versus Glu discrimination in cognate tRNA charging. 34-CUC-36 binds the anticodon-binding domain. Glutamate is a negatively charged amino acid with significant chemical character. No editing reactions are identified for GluRS-IB, consistent with the idea that glutamate is more readily discriminated by GluRS-IB than column 1 (Val, Leu, Ile, Met and Phe) and column 2 (Ala, Thr, Pro and Ser) amino acids that require cognate AARS enzymes that edit. Amino acids encoded in columns 3 and 4 have greater chemical character and less need for editing for error correction.

**Figure 29:**
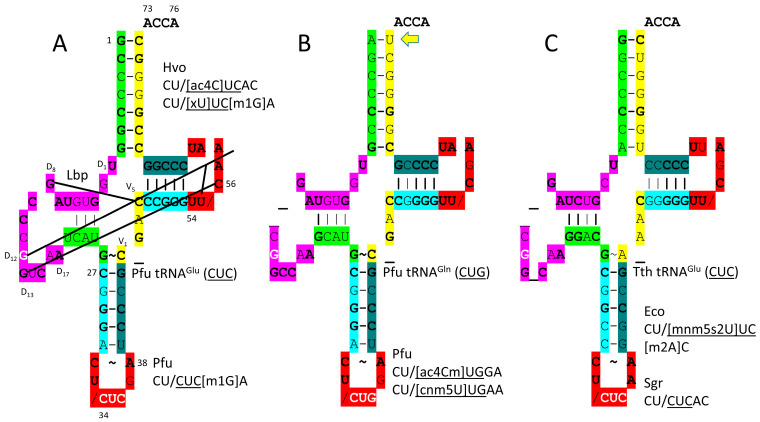
tRNA^Glu^ and tRNA^Gln^. (**A**) *P. furiosus* tRNA^Glu^ (CUC). (**B**) *P. furiosus* tRNA^Gln^ (CUG). (**C**) *T. thermophilus* tRNA^Glu^ (CUC). Sgr for *S. griseus.* xU indicates an unknown 5-carbon U34 modification to suppress superwobbling.

tRNA^Glu^ and tRNA^Gln^ are compared in Figure 29. In *P. furiosus*, tRNA^Glu^ (CUC) (Figure 29A) is very close to the primordial tRNA sequence. The acceptor stem of tRNA^Glu^ (CUC) varies by only 2 bp from tRNA^Pri^. The D loop has the sequence D_1_-UGGUGUAGCCCGGUCAA-D_17_, differing from the primordial sequence in six positions but including no deletions relative to tRNA^Pri^. By contrast, tRNA^Gln^ (CUG) has two deletions from the primordial sequence in the D loop (Figure 29B). For tRNA^Glu^ (CUC), the anticodon stem matches tRNA^Pri^ in 3 of 5 bp. For tRNA^Glu^ (CUC) and tRNA^Gln^ (CUG), the V loop has the sequence C_GAC, which matches the primordial sequence of CCGCC in three positions. Also, the V loop sequence C_GAC is found in tRNA^Asp^ (GUC) (Figure 25A), indicating that tRNA^Glu^ (CUC) may be derived from tRNA^Asp^, as might be expected. The T stem–loop–stem of tRNA^Glu^ (CUC) is a perfect match to the primordial sequence. For tRNA^Gln^ (CUG), the T stem–loop–stem sequence is slightly altered relative to tRNA^Pri^. We note that tRNA^Gln^ (CUG) has an unusual 1A=72U pair that is expected to separate more easily than 1G=72C in tRNA^Glu^ (CUC) and many other *P. furiosus* tRNAs. Melting the 1A=72U pair in tRNA^Gln^ (CUG) should contribute to discriminator function (i.e., in pre-life and until eukaryogenesis, for the Glu to Gln amidotransferase). In *T. thermophilus*, the tRNA^Glu^ (CUC) is similar to *P. furiosus* but more derived from the root sequence, as expected. As mentioned above, tRNA^Asp^, tRNA^Glu^ and tRNA^Gln^ are closely related sequences in *P. furiosus*.

### 4.15. LysRS-IB

Currently, no suitable demonstration structure of archaeal LysRS-IB-tRNA^Lys^ is available. Because of homology, we assume the structure would be similar to the image of GluRS-IB-tRNA^Glu^ (CUC) (Figure 28). LysRS-IB in Archaea appears to be the oldest LysRS. LysRS-IIB in Bacteria appears to be derived from AspRS-IIB, as a bacterial innovation. In Archaea, GluRS-IB, LysRS-IB (archaeal type) and GlnRS-IB (from Eukarya) are closely related AARS enzymes.

**Figure 30:**
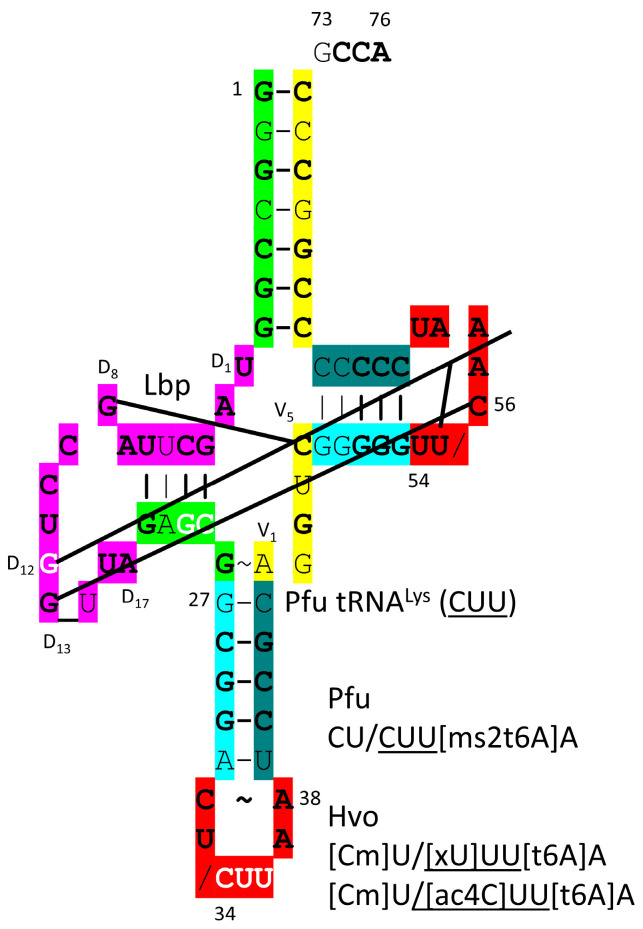
*P. furiosus* tRNA^Lys^ (CUU). xU is an unidentified 5-carbon U34 modification to suppress superwobbling.

In Figure 30, a *P. furiosus* tRNA^Lys^ (CUU) is shown. The acceptor stem sequence varies in only 2 bp from the primordial tRNA sequence. The D loop has the sequence D_1_-UAGCUUAGCCUGG_UUA-D_17_, differing in three positions from the primordial sequence, including a single base deletion relative to tRNA^Pri^. The 5′-As* sequence GAGCG differs in only one position from the typical primordial sequence GGGCG. The anticodon stem matches tRNA^Pri^ in 3 of 5 bp. The type I V loop sequence AGGUC is typical. The T stem–loop–stem matches the primordial sequence in all but 2 stem bp. The modifications of the anticodon loop are as expected. In *P. furiosus*, tRNA^Lys^ is most similar to tRNA^Phe^ and somewhat similar to tRNA^Arg^. Lysine and arginine are positively charged amino acids and may both be derived from ornithine by pre-life metabolism [77].

### 4.16. AlaRS-IID

Alanine is proposed to be the founding amino acid for column 2 of the genetic code. It is hypothesized that AlaRS-IID may have replaced a now extinct AlaRS-IIA before LUCA, so there may be no sequence record of an earlier AlaRS-IIA. Homology comparing a IID and a IIA AARS is difficult to discern, so these are very different enzymes. The reason for the replacement may be to discriminate alanine, serine, threonine and proline. Column 2 of the genetic code includes SerRS-IIA, ThrRS-IIA and ProRS-IIA, indicating evolution in code columns. Column 2 of the code is divided into all 4-codon boxes.

**Figure 31:**
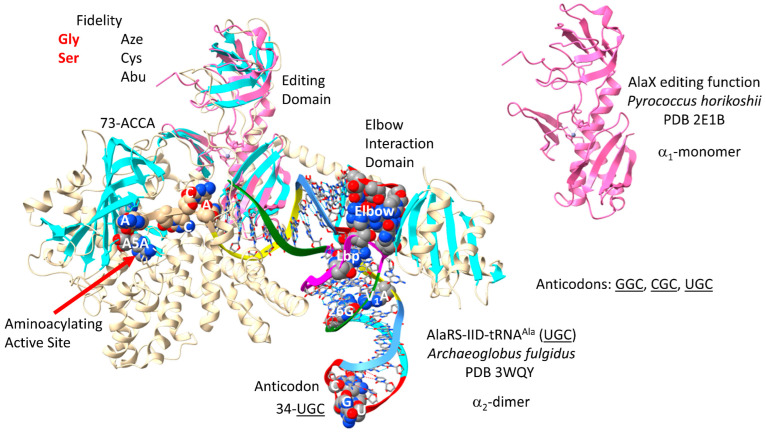
AlaRS-IID-tRNA^Ala^ (UGC) of *A. fulgidus*. The AlaX protein of *P. horikoshii* (pink) is also shown and overlaid on the *A. fulgidus* structure to locate the editing active site. No contact is made by AlaRS-IID with the tRNA^Ala^ anticodon loop.

AlaRS-IID-tRNA^Ala^ (UGC) of Archaeon *Archaeoglobus fulgidus* is shown in Figure 31 [93]. AlaRS-IID functions as an α_2_-dimer. This image is of only half of the protein. Interestingly, although alanine is located at a 4-codon sector, AlaRS-IID makes no contact with the tRNA^Ala^ anticodon loop. AlaRS-IID makes extensive elbow contacts, which may indicate tRNA^Ala^ distortion and allosteric effects of tRNA^Ala^ binding. 73-ACCA-76 penetrates the aminoacylating active site, which is also identified by the surface of antiparallel β-sheets and A5A reaction intermediate analogue binding. AlaRS-IID includes a separate editing active site that removes non-cognate azetidine-2-carboxylic acid, cysteine and α-aminobutyrate from tRNA^Ala^. The aminoacylating active site of AlaRS-IID limits non-cognate glycine and serine attachment to tRNA^Ala^. In Archaea, a separate AlaX editing enzyme is also present that can remove non-cognate amino acids from tRNA^Ala^ [94]. In the image shown, AlaX is light pink and was overlaid with the AlaRS-IID structure to locate the editing active site domain. AlaX may partly compensate for the lack of anticodon recognition by AlaRS-IID. Ala is a small hydrophobic amino acid with little chemical character, which may explain why AlaRS-IID has editing functions, including the trans AlaX editing function.

**Figure 32:**
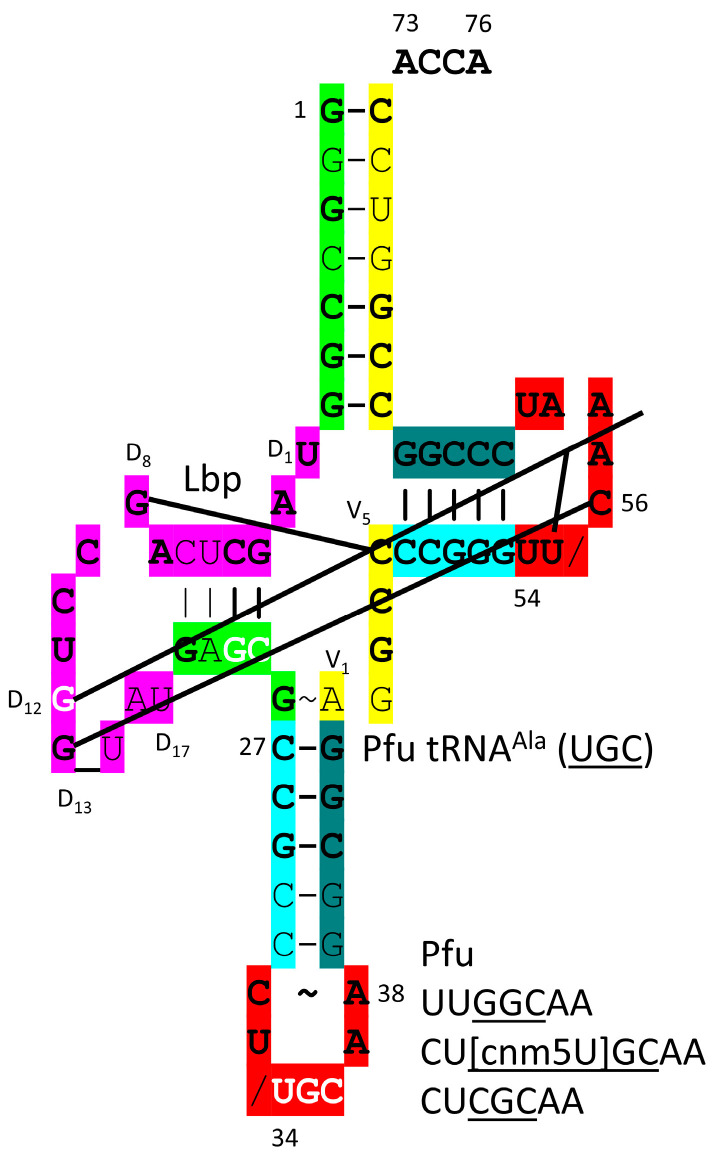
*P. furiosus* tRNA^Ala^ (UGC).

The *P. furiosus* tRNA^Ala^ (UGC) is shown in Figure 32. The acceptor stem of tRNA^Ala^ (UGC) varies in only 2 bp from the primordial tRNA sequence. The D loop has the sequence D_1_-UAGCUCAGCCUGG_UAU-D_17_, matching the primordial sequence in all but six positions, with a single base deleted from tRNA^Pri^. The 5′-As* sequence has the sequence GAGCG versus the typical GGGCG. The anticodon stem matches tRNA^Pri^ in 3 of 5 bp. The V loop has the sequence AGGCC versus CCGCC for the primordial tRNA and AGGUC for the typical tRNA. The T stem–loop–stem matches the primordial tRNA. The *P. furiosus* tRNA^Ala^ has the appearance of an ancient tRNA, consistent with GADV being the first four amino acids in the code. As noted above, the *P. furiosus* tRNA^Ala^ is similar in sequence to tRNA^Val^, consistent with alanine and valine being early additions to the code.

### 4.17. PheRS-IIC

It is hypothesized that aromatic amino acids (Phe, Tyr and Trp) entered the genetic code as some of the last amino acids added, in the disfavored row 1 (tRNA-36A) [95]. It is suggested that row 1 (tRNA-36A) was disfavored because, initially, both the tRNA-34 and tRNA-36 positions of the anticodon were wobble positions. During the evolution of the code, tRNA-34 remained a wobble position, but wobbling at tRNA-36 was suppressed. Wobbling at tRNA-36 was suppressed, in part, by modification of tRNA-37. Notably, if tRNA-36U is present, generally, tRNA-37A is modified (i.e., t6A or hn6A). If tRNA-36A is present, generally, tRNA-37G is modified (i.e., m1G). tRNA-37t6A may be more effective at suppressing tRNA-36U wobbling compared to the efficacy of tRNA-37m1G at suppressing tRNA-36A wobbling. In contrast to tRNA-36, tRNA-34 remained a wobble position. For one thing, the modification of tRNA-33U cannot alter tRNA-34 reading, because tRNA-33U is on the opposite side of the anticodon loop U-turn. At the base of code evolution, tRNA-33 is always U. Also, tRNA-35 is a Watson–Crick position that cannot be modified in any way that interferes with coding. In evolution, tRNA-34 wobbling could not be suppressed.

The following model is proposed for PheRS evolution. The initial PheRS may have been PheRS-IC derived distantly from a primitive ArgRS-IA or GluRS-IB. As TyrRS-IC and TrpRS-IC differentiated, there was insufficient discrimination between phenylalanine and tyrosine. PheRS-IC was then replaced by PheRS-IIC, before LUCA, leaving no sequence trace of PheRS-IC, except for TyrRS-IC and TrpRS-IC.

**Figure 33:**
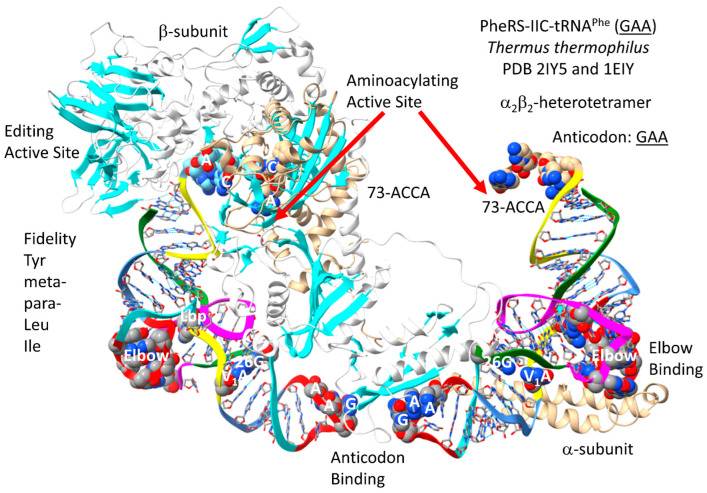
PheRS-IIC-tRNA^Phe^ (GAA) from *T. thermophilus*. Both tRNA^Phe^ (GAA) are shown to indicate all relevant PheRS-IIC-tRNA^Phe^ (GAA) contacts.

A detail of PheRS-IIC-tRNA^Phe^ (GAA) from *T. thermophilus* is shown in Figure 33 [96,97]. PheRS-IIC in *T. thermophilus* functions as an α_2_β_2_-dimer, which is also the archaeal form. Only one αβ-unit is shown. To observe the relevant tRNA contacts, both tRNA^Phe^ (GAA) were visualized. The aminoacylating active site is a surface of antiparallel β-sheets in the α-subunit. 73-ACCA-76 penetrates to the aminoacylating active sites. The separate editing active site is within the β-subunit. PheRS-IIC removes non-cognate tyrosine, meta- and para-substituted phenylalanine derivatives, leucine and isoleucine from tRNA^Phe^ (GAA). An extrusion of the α-subunit makes elbow contact. An extrusion of the β-subunit makes anticodon contacts.

**Figure 34:**
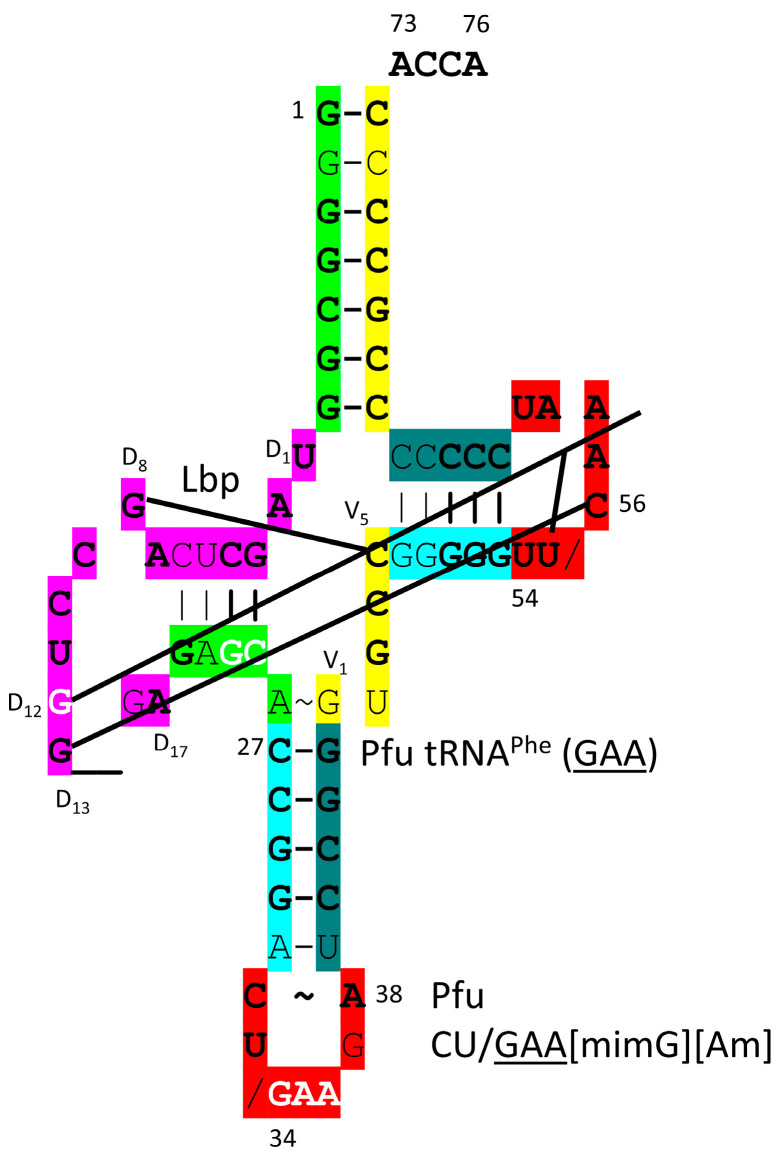
*P. furiosus* tRNA^Phe^ (GAA).

The *P. furiosus* tRNA^Phe^ (GAA) is shown in Figure 34. The acceptor stem matches the primordial sequence in all but a single base pair. The D loop has the sequence D_1_-UAGCUCAGCCUGG__GA-D_17_, matching the primordial sequence in all but five positions, with two bases deleted relative to tRNA^Pri^. The 5′-As* sequence GAGCA matches the primordial sequence GGGCG in three positions. The anticodon stem matches the primordial sequence in 4 of 5 positions. The V loop sequence GUGCC matches primordial CCGCC in three positions. The T stem–loop–stem is very similar to the primordial sequence. Interestingly, tRNA^Phe^ (GAA) appears to be a relatively early tRNA, although phenylalanine appears to be a later entry into the code. In *P. furiosus*, tRNA^Phe^ (GAA) is closely related in sequence to tRNA^Lys^ (UUU) and (CUU).

### 4.18. TyrRS-IC

**Figure 35:**
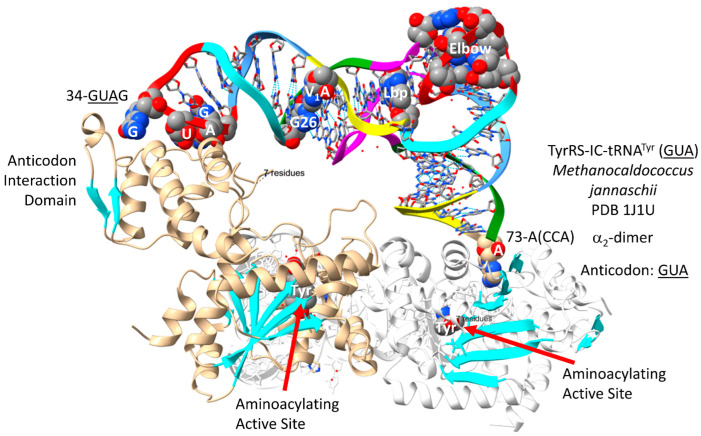
TyrRS-IC-tRNA^Tyr^ (GUA) of *M. jannaschii*.

It is hypothesized that aromatic amino acids were a late addition to the genetic code along the disfavored row 1 (tRNA-36A). In evolution, TyrRS-IC and TrpRS-IC may be derived from a primitive ArgRS-IA or GluRS-IB. In contrast to most class I AARSs, which are α_1_-monomers, TyrRS-IC and TrpRS-IC are obligate α_2_-dimers, with the anticodon-binding and the aminoacylating active site for a single cognate tRNA in separate α-subunits. The TyrRS-IC-tRNA^Tyr^ (GUA) from Archaeon *Methanocaldococcus jannaschii* is shown in Figure 35 [98]. Aminoacylating active sites are at the C-terminal ends of a set of parallel β-sheets. Tyrosine is bound at the aminoacylating active sites. 73-A(CCA)-76 is partly disordered in the structure. The anticodon 34-GUA-36 contacts the anticodon interaction domain. One tRNA^Tyr^ (GUA) is white and mostly obscured in this image.

**Figure 36:**
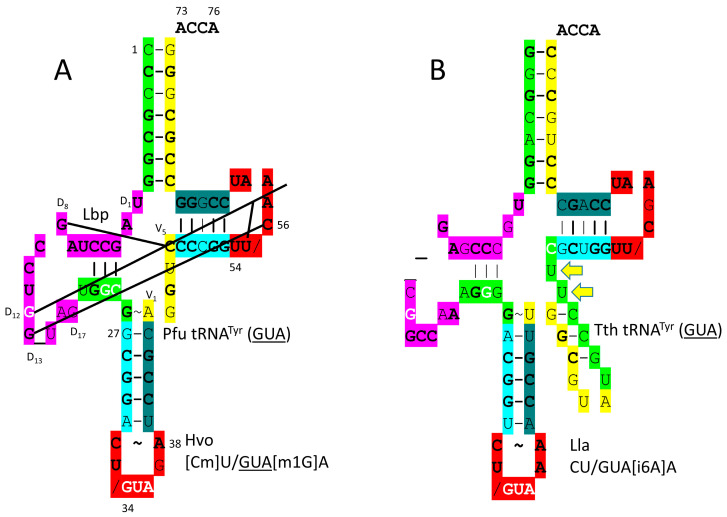
tRNA^Tyr^ (GUA). (**A**) Archaeal *P. furiosus* tRNA^Tyr^ (GUA) (type I). (**B**) Bacterial *T. thermophilus* tRNA^Tyr^ (GUA) (type II). Lla for *Lactobacillus lactis*. In Bacteria, type II tRNA^Tyr^ has two unpaired bases separating the 3′-V stem from the Levitt base.

In Figure 36A, the *P. furiosus* tRNA^Tyr^ (GUA) is shown. The acceptor stem matches the primordial tRNA in all but two base pairs. The D loop sequence is D_1_-UAGCCUAGCCUGG_UAG-D_17_, matching the primordial sequence in all but four positions, with a single base deleted relative to tRNA^Pri^. Consistent with the three 31 nt minihelix tRNA evolution theorem, the D loop sequence begins with two perfect UAGCC repeats. The 5′-As* sequence is UGGCG, matching the typical primordial sequence GGGCG in all but a single base. The anticodon stem matches tRNA^Pri^ in 3 of 5 bp. The type I V loop is the typical AGGUC. The T stem–loop–stem matches the primordial sequence in all but a single stem base pair. In Archaea, tRNA^Tyr^ (GUA) is a type I tRNA. In Bacteria, by contrast, tRNA^Tyr^ (GUA) is a type II tRNA [11] (Figure 36B). The difference appears to be a bacterial innovation.

### 4.19. TrpRS-IC

**Figure 37:**
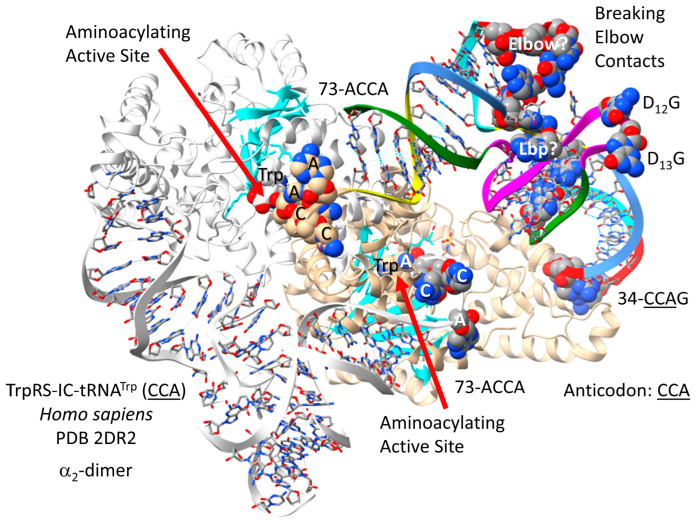
TrpRS-IC-tRNA^Trp^ (CCA) from *H. sapiens*.

TrpRS-IC [99] is a very similar enzyme to TyrRS-IC. In the H. sapiens TrpRS-IC-tRNA^Trp^ (CCA) structure (Figure 37), 73-ACCA-76 enters the aminoacylating active site, where tryptophan binds. A set of parallel β-sheets approach the aminoacylating active site. There are substantial allosteric effects on tRNA^Trp^ (CCA) from TrpRS-IC binding. Elbow contacts between the D loop (D_12_-GG-D_13_) and the T loop (54-UU/CAA-58) are broken. The Levitt bp is also disrupted. Deformability of tRNA^Trp^ (CCA) may contribute to cognate tryptophan charging. Tryptophan is in a 1-codon box in the code, which is generally not allowed. Tryptophan can be in a 1-codon box because Trp shares a 2-codon box with a stop codon (UGA), which does not utilize a tRNA but rather is recognized by a protein release factor binding to the UGA stop codon on the mRNA on the ribosome. Methionine is also in a 1-codon box that is shared with isoleucine (anticodon CAU). In this case, different wobble 34C modifications explain how translational accuracy is maintained, and the UAU Ile anticodon is generally not utilized.

**Figure 38:**
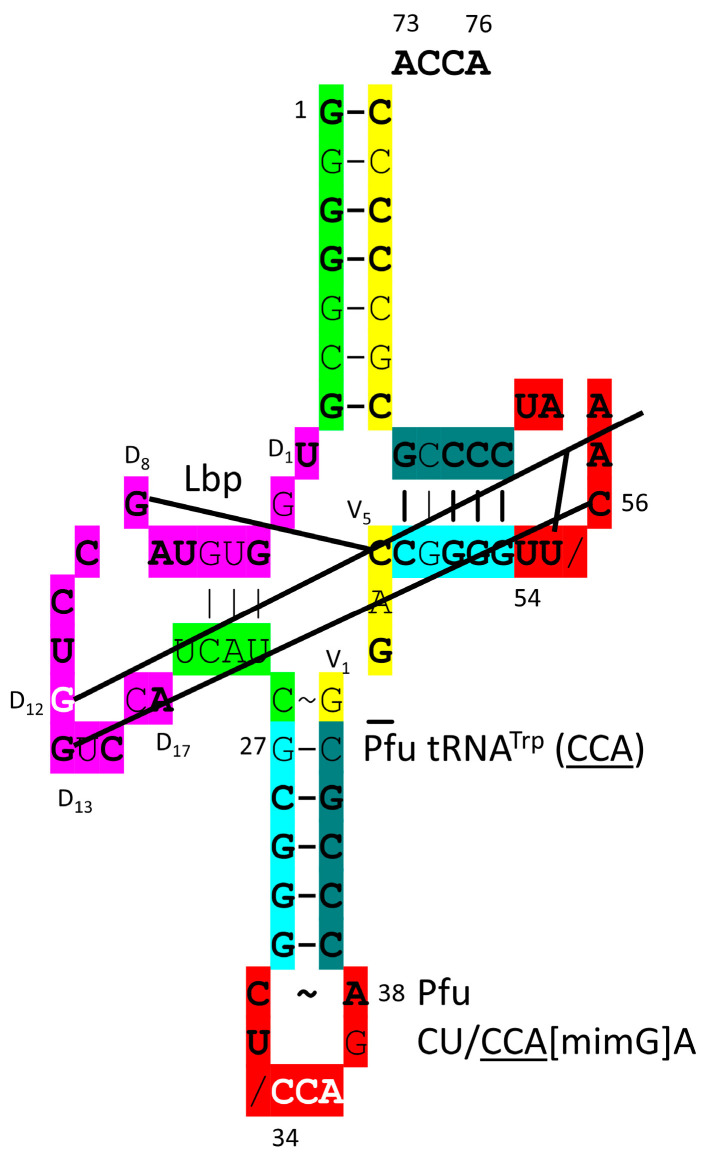
*P. furiosus* tRNA^Trp^ (CCA).

The *P. furiosus* tRNA^Trp^ (CCA) is shown in Figure 38. The acceptor stem matches the primordial tRNA sequence at 4 bp. The tRNA has a D loop with the sequence D_1_-UGGUGUAGCCUGGUCCA-D_17_, matching the primordial sequence in all but five positions and including no deletions from tRNA^Pri^. The anticodon stem matches tRNA^Pri^ in 4 of 5 bp. The T stem–loop–stem matches the primordial tRNA sequence in all but one stem base pair. In *P. furiosus*, tRNA^Trp^ (CCA) is similar to tRNA^Pro^ (GGG, CGG and UGG).

## 5. The Genetic Code

**Figure 39:**
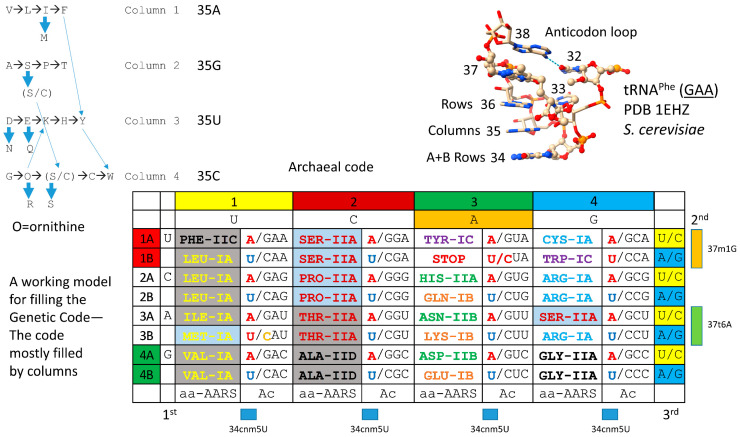
A model for the evolution of the first code. A codon–anticodon table is shown with a maximum complexity of 32 assignments, as in tRNA. Codons are shown in sectors marked 1st, 2nd and 3rd. Anticodons (Ac) are indicated (i.e., 34-[A/G]AA-36). Anticodons that are not utilized are shown in red letters. No tRNA matches stop codons (UAA, UAG, and UGA). Blue 34U indicates a modification to limit superwobbling, such as 34cnm5U. As indicated above, some exceptions have been noted, but wobble 5C-U modifications to suppress superwobbling may have been universal at the inception of the first code. 37m1G is associated with 36A. 37t6A is associated with 36U. Column 1, row 3B, 34C modifications (orange) discriminate Ile and Met. The genetic code evolved primarily in columns, as indicated in the model. In column 1, ValRS-IA, LeuRS-IA, IleRS-IA and MetRS-IA are closely related enzymes (yellow type). In column 2, SerRS-IIA, ProRS-IIA and ThrRS-IIA are closely related enzymes (red type). In column 3, AspRS-IIB, AsnRS-IIB and HisRS-IIA are closely related (green type), and GluRS-IB, LysRS-IB (in Archaea) and GlnRS-IB (a eukaryotic innovation) are closely related (orange type). In column 4, ArgRS-IA and CysRS-IA are closely related. In row 1, TyrRS-IC and TrpRS-IC are closely related. In the anticodon loop image, modified bases are indicated in ball-and-stick representation. A similar figure was previously published and is republished here with permission [15,16].

**Figure 40:**
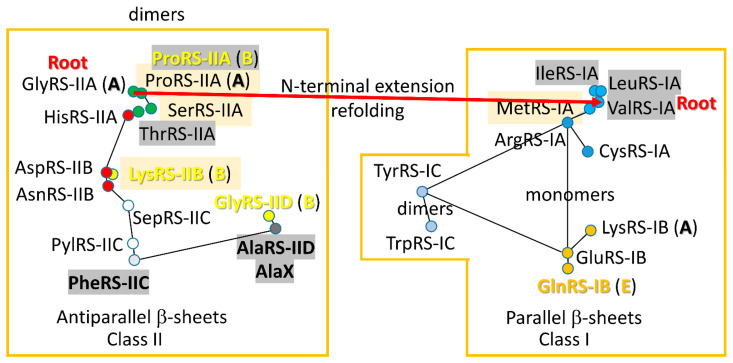
Evolution of AARS enzymes. Phyre 2 homology scoring mostly to *P. furiosus* AARS sequences was used to draw the class II and class I AARS maps. GlyRS-IIA is homologous to ValRS-IA and IleRS-IA by sequence as indicated by the red arrow. AARSs with separate editing active sites are shaded gray. AARS that have editing reactions only in their aminoacylating active sites are shaded pale yellow. Bacterial innovations are indicated (B). Archaeal-type AARSs are indicated (A). GlnRS-IB was a eukaryotic innovation (E). GlyRS-IIA appears to be the root of all class II and class I AARSs. A primitive ValRS-IA appears to be the root of all class I AARSs. PheRS-IIC and AlaRS-IID are in bold because these enzymes may have replaced PheRS-IC and AlaRS-IIA before LUCA. Sep stands for o-phosphoserine. Pyl stands for pyrrolysine.

**Figure 41:**
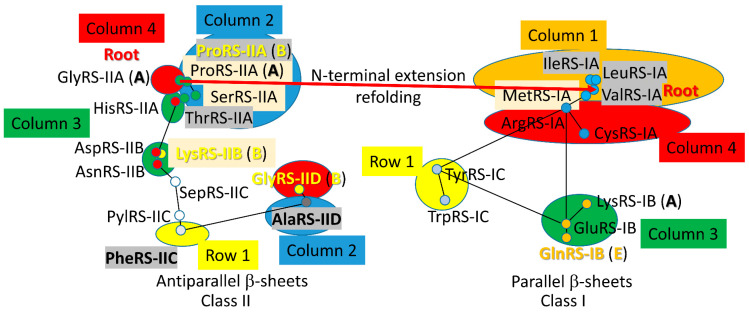
Relationship of AARS enzymes and the genetic code. Column 1 amino acids and AARSs are on an orange background. Column 2 amino acids and AARSs are on a blue background. Column 3 amino acids and AARSs are on a green background. Column 4 amino acids and AARSs are on a red background. Row 1 amino acids and AARSs are on a yellow background. Other indications are as in Figure 40.

A model for the evolution of the first genetic code is shown in Figure 39 [15,16,17,18,64]. Much of the data supporting this model are summarized in Figure 40 and Figure 41. The code is represented as a codon–anticodon table with a complexity of 32 assignments, rather than 64 assignments. Because of code degeneracy, 32 assignments (2 × 4 × 4) is the maximum complexity of the genetic code in tRNA, because a wobble position (tRNA-34) has only purine versus pyrimidine resolution. The code is highly ordered. Most of the evolution is in code columns. The history of the evolution of AARS enzymes (summarized below) relates a fairly straightforward story of evolution of the code. The genetic code is simpler and more ancient in Archaea. The code is more innovated in Bacteria and Eukarya [61].

The model for the evolution of the genetic code relies on the solution of tRNA evolution [15,30,31]. For tRNA, the orderly mechanism of tRNA assembly and tRNA root sequences is known (Figure 1 and Figure 2). tRNA evolved according to the three 31 nt minihelix tRNA evolution theorem. Based on sequences, this is much more of a theorem (a proven theory) than a conjecture or hypothesis or model. The original tRNA sequence was 100% RNA repeats (GCG, CGC and UAGCC) and inverted repeats (~CCGGG_CU/GCCAA_CCCGG; _ separates stems and loops; / indicates a U-turn; the only sequence ambiguity is in the anticodon GCC, which has since been scrambled in coding). Because the initial tRNA was so highly ordered, tRNA evolution was solved by inspection as a simple puzzle. ACCA-Gly was ligated at the tRNA 3′-end to synthesize polyglycine. There is no “chicken and egg” problem in the evolution of the genetic code, because the code initially evolved to synthesize polyglycine and subsequently advanced to encode GADV polymers. The code did not need foresight of its evolving role in encoding RNA sequence-dependent proteins.

The evolution of the code makes the best sense when viewed by code columns. The model for filling the code might be the following: G (polyglycine) evolved to GADV, which evolved to GADVLSER, which evolved to GADVLSERNCQ, which evolved to GADVLSERNCQPTIMHK, which evolved to GADVLSERNCQPTIMHKFYW [15,16,100]. At about the 11 amino acid stage, GADVLSERNCQ might be expected to support synthesis of the first sequence-dependent proteins. Dividing the evolutionary history into code columns makes the best sense (Figure 39). NCQ, and possibly other amino acids, were added through tRNA-linked chemistry, giving insights into a major mechanism for RNA-linked pre-life metabolism.

### 5.1. Column 1

In column 1 (tRNA-35A), valine may be the founding amino acid. It appears that valine (tRNA-36C) goes to leucine, which goes to isoleucine, which goes to methionine. Phenylalanine is added last along the disfavored row 1 (tRNA-36A). In metabolism, valine can be converted to leucine in five steps. Thus, leucine may have been initially added to the code by tRNA-linked chemistry. Val-tRNA^Val^ may have been converted to Leu-tRNA^Leu^ in several steps, either supported by ribozymes or by the first protein catalysts. We posit that tRNA-linked and RNA-linked chemistry were very ancient and were fundamental to the evolution of pre-life metabolism and the genetic code. Notably, the evolution of tRNA and the divergence of tRNAomes is a story of RNA–amino acid and RNA–protein evolution [43]. In the first code, tRNA^Val^ is type I and tRNA^Leu^ is type II, and type I tRNA was processed from a primitive type II tRNA (Figure 1 and Figure 2). During early tRNAome assembly, tRNAs may have been mixed type I and type II, and the mixtures may have been sorted later by selection to construct the first code. In Archaea, only leucine and serine utilize type II tRNAs. In Bacteria, tyrosine, leucine and serine utilize type II tRNAs [11]. The number of amino acids supported by type II tRNAs was limited by the number of allowed trajectory set points of the type II V arm.

In Archaea, type II tRNAs encoding leucine and serine were selected to substitute longer V arms for anticodon loop recognition by their cognate AARSs, because 5 tRNA^Leu^ and 4 tRNA^Ser^ were necessary [11]. Isoleucine was the next amino acid to enter column 1. Neither valine nor leucine can be converted to isoleucine. Threonine (tRNA-36U), which borders isoleucine (tRNA-36U) in column 2 of the code, however, can be converted to isoleucine. It may be that Thr-tRNA^Thr^ evolved to Ile-tRNA^Ile^ via tRNA-linked chemistry. It is hypothesized that isoleucine briefly occupied a 4-codon sector of the code that was invaded by methionine. It appears that tRNA^Ile^ may have evolved to tRNA^Met^. In *P. furiosus*, tRNA^Ile^ and tRNA^Met^ are similar. It appears that methionine invaded a 4-codon isoleucine sector. At the base of the code evolution, the anticodon UAU was eliminated. Without modification, UAU would cause confusion between encoding isoleucine and methionine. Both the initiator and elongator tRNA^Met^ (CAU) evolved. The initiator tRNA^Met^ (CAU) is unmodified at 34C. The elongator tRNA^Met^ (CAU) utilizes the 34Cm modification. Phenylalanine was added to the code late (described below). In Archaea, 34agm2CAU (agm2C for agmatidine) encodes isoleucine. In Bacteria, 34k2CAU (k2C stands for lysidine) encodes isoleucine.

### 5.2. Column 2

Column 2 (tRNA-35G) of the code appears to have evolved from alanine to serine and then to proline and threonine. Serine appears to have jumped from column 2 to column 4 of the genetic code, perhaps, in part, to obtain a more favorable anticodon (tRNA-35C appears to be favored over tRNA-35G). Alanine can be converted to serine in several steps, so Ala-tRNA^Ala^ may have evolved to Ser-tRNA^Ser^. If this is the case, however, type I tRNA^Ser^ was probably replaced from type II tRNA^Leu^. From sequences, it appears that type II tRNA^Ser^ was derived from type II tRNA^Leu^. Serine is a special case, because serine is the only amino acid that appears to jump columns in the establishment of the genetic code. Also, serine can be converted to cysteine by tRNA-linked chemistry [82,101,102]. It is hypothesized that the conversion of serine to cysteine may relate to the jumping of serine from column 2 to column 4 of the code. To establish the standard code, cysteine landed in column 4, the disfavored row 1. Cysteine, however, must have entered the code earlier, perhaps linked to serine within an expanded serine sector. In proteins, cysteine is necessary for Zn binding, which was required for the first protein folding. AARS enzymes are an example of the first proteins, coevolved with the genetic code, whose folding depended on cysteine binding Zn. Cysteine may have occupied the disfavored row 1 (tRNA-36A) late in the evolution of the code. Serine may have displaced cysteine in row 3, column 4, where serine now resides. Serine appears to have jumped columns by invading and splitting an expanded arginine sector.

Because serine utilizes a type II tRNA^Ser^, and because SerRS-IIA lacks tRNA^Ser^ anticodon recognition, these features may have facilitated serine or serine/cysteine jumping in the evolution of the code. A change of Ser/Cys-tRNA^Ser/Cys^ (GGU)→Ser/Cys-tRNA^Ser/Cys^ (GCU) could account for serine jumping columns. GGU becomes a threonine anticodon, indicating that the threonine 4-codon sector (column 2, row 3) displaced serine from an expanded serine sector. Threonine and serine are chemically related amino acids. Proline also appears to have displaced serine to form a 4-codon sector (column 2, row 2).

It is hypothesized that the first AlaRS may have been an AlaRS-IIA, from which column 2 SerRS-IIA, ThrRS-IIA and ProRS-IIA were derived. As the genetic code was built up, however, we posit that AlaRS-IIA was replaced by AlaRS-IID. The proposed replacement is analogous to the replacement of archaeal-type GlyRS-IIA by GlyRS-IID in more derived Bacteria. If the AlaRS-IIA-to-AlaRS-IID replacement event was prior to LUCA, there may now be no sequence record of AlaRS-IIA. The AlaRS-IID innovation helped discriminate neutral amino acids, alanine, serine, threonine and proline. AlaRS-IID has editing functions, and AlaRS-IID has a separate editing domain. In ancient organisms, AlaRS-IID utilizes the AlaX editing protein to support accuracy (Figure 31). The AlaRS-IID aminoacylating active site also has editing functions. The AlaX protein may partially compensate for the lack of AlaRS-IID anticodon loop recognition. Alanine is in a 4-codon box, but AlaRS-IID does not utilize the tRNA^Ala^ anticodon loop as a determinant for accurate charging.

### 5.3. Column 3

Column 3 is the most innovated column in the code, encoding the most amino acids. Notably, column 3 is broken into all 2-codon sectors. It is hypothesized that column 3 may have been sectored by a slightly different mechanism compared to columns 1, 2 and 4. We suggest that early in code evolution both tRNA-34 and tRNA-36 were wobble positions, but only a single wobble position could be utilized at a time. According to this view, columns 1, 2 and 4 primarily utilized Watson–Crick 35 and wobble 36. Column 3 primarily utilized Watson–Crick 35 and wobble 34. tRNA-35 was always the easiest to read because this is the central base in the anticodon. In a wobble position, only purine–pyrimidine discrimination is achieved, so only two possible code assignments are obtained. In such a scenario, the complexity of the evolving code would be 2 × 4 or 4 × 2 or 8 amino acids, depending on the wobble position (tRNA-34 or tRNA-36). Because of tRNA-linked chemistry adding NCQ, the limited code probably expanded to 11 amino acids (GADVLSER expanded to GADVLSERNCQ). We posit that the evolution of the genetic code was hung up at 8 or 11 amino acids until wobbling at tRNA-36 could be suppressed. Wobbling at tRNA-36 was suppressed, in part, by modifications at tRNA-37. tRNA-37G modifications (i.e., m1G) were used to read tRNA-36A. tRNA-37A modifications (i.e., t6A) were used to read tRNA-36U. It appears that wobbling at tRNA-36U was more readily suppressed than wobbling at tRNA-36A. Notably, 37t6A to suppress 36U is a more dramatic modification than 37m1G to suppress 36A. In the evolution of the code, row 3 (tRNA-36U) of the genetic code appears to have been more favorable than row 1 (tRNA-36A).

Column 3 appears to have first encoded aspartic acid. The chemically related glutamic acid may then have invaded the B rows. This resulted in a Glu-Asp-Glu-Asp-Glu-Asp (column 3, row 4B-4A-3B-3A-2B-2A) pattern (Figure 39). Asparagine displaced aspartic acid in column 3, row 3A. Histidine displaced aspartic acid in column 3, row 2A. Lysine displaced glutamate in column 3, row 3B. Glutamine displaced glutamate in column 3, row 2B. Stop codons and tyrosine were added late across the disfavored row 1.

As soon as aspartic acid and glutamate entered the code, tRNA-linked chemistry generated asparagine and glutamine [87,90,91,103,104]. Serine can be converted to cysteine by tRNA-linked chemistry [82,101,102]. The 8 amino acid code (i.e., GADVLSER), therefore, rapidly evolved to an 11 amino acid code (GADVLSERNCQ) by tRNA-linked chemistry. The 11 amino acid code appears to be sufficient to generate the first RNA sequence-dependent proteins.

### 5.4. Column 4

Column 4 (tRNA-35C) appears to be the most favored code column. Glycine appears to occupy the most favored sector of the genetic code (tRNA-35C, tRNA-36C). It is hypothesized that glycine is the founding amino acid in the code. GADV, the four simplest and probably the four initial encoded amino acids, occupy the most favored row of the genetic code (tRNA-36C) [65,66,67,68,105,106]. These observations are consistent with glycine being the first encoded amino acid [19,20]. In *P. furiosus*, tRNA^Gly^ is the most similar to tRNA^Pri^, indicating that glycine may have been the first encoded amino acid [21]. GlyRS-IIA appears to be the root for all class II and class I AARSs. Glycine is the smallest and the most flexible amino acid. It is very likely that glycine was the founding amino acid in the evolution of the genetic code.

In contrast with glycine, arginine, which is also in column 4, is a complex amino acid. It is hypothesized that ornithine may have been the founding positively charged amino acid [77]. Ornithine can be converted to arginine in two metabolic steps. In some Archaea and Bacteria, ornithine can be converted to lysine by the α-aminoadipate pathway [107,108,109,110,111,112]. Thus, arginine and lysine may have entered the genetic code through tRNA-linked reactions: Orn-tRNA^Orn^ evolved to Arg-tRNA^Arg^ (column 4), and Orn-tRNA^Orn^ (UCU, CCU) (column 4) evolved to Lys-tRNA^Lys^ (UUU, CUU) (column 3).

ArgRS-IA is the closest relative of CysRS-IA, indicating how cysteine may have evolved to its current placement in column 4, row 1A, of the code. In *P. furiosus*, only tRNA^Thr^ (column 2) and tRNA^Cys^ (column 4) utilize the discriminator base 73U. tRNA^Thr^ and tRNA^Cys^ are similar in sequence in *P. furiosus*.

### 5.5. Disfavored Row 1

The disfavored row 1 of the genetic code appears to have been sectored last. Phenylalanine, tyrosine and tryptophan are complex aromatic amino acids. It has been hypothesized that, initially, phenylalanine spread across row 1, utilizing a primitive PheRS-IC. From PheRS-IC, both TyrRS-IC and TrpRS-IC may have been derived. To suppress translation errors, it is hypothesized that PheRS-IC was replaced by PheRS-IIC before LUCA. There now appears to be no sequence trace of PheRS-IC. PheRS-IIC has a separate editing active site to suppress non-cognate charging with tyrosine and other amino acids. In Bacteria, tRNA^Tyr^ (GUA) is a type II tRNA, but this is a bacterial innovation, perhaps, to suppress translation errors (i.e., enhancing the discrimination of Phe and Tyr). Amino acids that differ only in a hydroxyl group are difficult for AARS enzymes to distinguish [36].

Serine appears to have ended up in column 2, row 1, from its particular chaotic history in genetic code evolution that may have involved the invasion of an expanded serine sector by threonine and proline. Cysteine may have ended up in column 4, row 1A, from the history of serine jumping from column 2 to column 4.

We conclude that a rational explanation can be provided for the placements of: (1) all amino acids; (2) class II and class I AARS enzymes; and (3) many tRNAs in the evolution of the genetic code. When this project was started, this outcome was not anticipated.

### 5.6. Stop Codons and Evolution of Translational Fidelity

Stop codons are located at column 3, row 1B, and column 4, row 1B. We consider it likely that stop codons were a late addition to the code. The evolution of the genetic code can be viewed as the evolution of intellectual property initially to support polypeptide polymer synthesis as a pre-life chemistry emulsifier and then progressing to cognate coding with the inception of complex life. According to this view, initially, in pre-life, long protein polymers and innovation in amino acid additions were selected over fidelity. Translational fidelity became ever more important, however, as the genetic code evolved and the system developed intellectual property in tRNAomes, AARSomes and the first proteins that were more strongly selected.

Initially, the code evolved to synthesize polyglycine and then GADV polymers as emulsifiers for metabolic reaction components and to coalesce the first protocells. As accurate coding became more strongly selected, the selective pressure was toward the evolution of fidelity mechanisms, such as editing mechanisms in AARS aminoacylating active sites and separate proofreading domains. Stop codons and frame maintenance are also fidelity mechanisms.

Amino acids with little chemical character are located at the left half of the genetic code (Val, Met, Ile, Leu, Phe, Ala, Thr, Pro, and Ser) (columns 1 and 2). These amino acids are charged to cognate tRNAs by AARS enzymes that edit either within separate proofreading active sites, within the aminoacylating active site, or both. Amino acids from the right half of the code have more chemical character (Glu, Asp, Lys, Asn, Gln, His, Tyr, Gly, Arg, Trp and Cys). Cognate charging of right-half amino acids (columns 3 and 4) generally does not require editing. Right-half amino acids have more chemical character (i.e., charge, hydrogen bonding, and metal binding (i.e., Cys)) that is used to support accurate charging of their cognate tRNA.

Initially in pre-life, stop codons were not as important as later in evolution, because making longer emulsifying polymers was more important than accurate stops. Also, because of RNA ligations for RNA replication, combinations of primitive protein reading frames were strongly selected to generate more complex first proteins and new functions. When sequences were fused out of frame, therefore, more complex proteins were initially synthesized using frame shifts, before translation frames evolved to be in phase. A primitive class I ValRS-IA evolved by ligation of an N-terminal-encoding RNA to a primitive class II GlyRS-IIA-encoding RNA. In the absence of hard stop codons, the initial ligation was not necessarily in phase. Initially, innovation was strongly valued over accuracy. Stop codons are read by protein release factors in mRNA. Protein release factors bind to stop codons in mRNA and effect the nascent protein release from the ribosome [113]. No tRNA is associated with stop codons and translation termination. In suppressor strains, a tRNA anticodon mutates into a stop codon to add an amino acid, somewhat inefficiently, in place of a stop.

## 6. Radiation of AARSomes

Much has been written about the radiations of AARSomes and individual AARS enzymes [36,37,114,115,116,117,118]. To our knowledge, however, others have not attempted to correlate the radiation of AARSomes to the structure of the first genetic code, as we attempt here. We do not claim that our effort will be the final word on this project. We present a working model that probably can be improved using emerging network analysis techniques. In support of what we have done, our analysis of AARSomes fits the structure of the genetic code very closely.

Figure 39, Figure 40 and Figure 41 document the evolution of the first genetic code. Figure 39 shows the ordered structure of the code, indicating the relatedness of AARS enzymes [15,16,17,18,64]. Most of the evolution is in code columns, as indicated in the model. Figure 40 relates the relationships among all class II and class I AARSs in the ancient Archaeon *P. furiosus*. Some AARSs missing in *P. furiosus* were supplied from other species, as appropriate. *P. furiosus* was selected because *P. furiosus* has an ancient tRNAome that is similar to that of LUCA [21]. It was assumed that the *P. furiosus* AARSome would also be similar to LUCA. Figure 40 was prepared, as previously described, using Phyre 2 homology scoring by structure and sequence [18,119]. The relatedness of GlyRS-IIA and ValRS-IA and IleRS-IA sequences has previously been demonstrated [15,16,17,18,64]. Figure 41 shows how the structure of the genetic code relates to the apparent lineages of AARS enzymes by correlating the map in Figure 39 to the pattern of AARS evolution shown in Figure 40. In Figure 41, AARS enzymes are assigned background colors that relate to the structure of the code shown in Figure 39. These three figures summarize an ordered model for AARS and genetic code evolution.

A primitive GlyRS-IIA appears to be the root of both the class II lineage and the class I lineage. Based on sequence homology, it is hypothesized that a primitive ValRS-IA was derived from a primitive GlyRS-IIA by ligation of an N-terminal-encoding RNA to a GlyRS-IIA-encoding RNA. In pre-life, the replication of RNAs required ribozyme ligases that generated long and complex RNAs and complex proteins very early in evolution. tRNA evolution required ligation and complementary replication to generate tRNA out of 31 nt minihelices. Attachment of the N-terminal-encoding RNA to the primitive GlyRS-IIA-encoding RNA altered the folding of the translated protein to a primitive ValRS-IA. Zn-binding motifs were important in folding the first AARS enzymes.

AARS enzymes were among the first proteins, coevolved with the genetic code. Without full tRNAomes and AARSomes, there is no standard code. It is hypothesized that sequence-dependent proteins emerged at about the 11 amino acid stage of code evolution (i.e., GADVLSERNCQ) (R may initially have been O (ornithine) that radiated to R and K). The 11 amino acid stage provides sufficient chemical diversity (i.e., flexibility, hydrophobicity, hydrogen bonding, and charge) to encode the first proteins. The addition of amino acids to the code improved protein structure and function until 20 amino acids and stops were encoded. As the code froze, adding additional amino acids became more of a liability because of the threat posed to translational accuracy. There is tension between innovation and error catastrophe. To prevent error catastrophe, fidelity mechanisms such as amino acid identity (chemical character) and editing by AARSs froze the code. Early in code evolution, innovation was more strongly selected. Late in code evolution, fidelity mechanisms evolved to protect the intellectual property that pre-biology and emerging biology had generated.

The model for AARS radiation (Figure 39, Figure 40 and Figure 41) is a working model. More advanced network and evolutionary analyses will be necessary to confirm or improve the model. To enhance tRNAome networks, alignments of tRNAs must be optimized in the D loop region and V loop region.

## 7. Evolution of Complex Life

The pathway to evolve complex life on Earth, supported by a genetic adapter and genetic code, is mostly elucidated [15,30]. Once the genetic code arose, all features of complex life and biodiversity became possible. This solution is embedded in the sequence of tRNA^Pri^ and in the order of assembly of the genetic code. tRNA was formed from GCG, CGC and UAGCC repeats and the inverted repeats (i.e., ~CCGGG_CU/GCCAA_CCCGG). tRNA evolved from ligation of three 31 nt minihelices of mostly known sequence (GCGGCGG_UAGCC_UAGCCUA_GCCUA_CCGCCGC and ~GCGGCGG_CCGGG_CU/GCCAA_CCCGG_CCGCCGC). ACCA-Gly was ligated to various RNAs, including tRNAs, to synthesize polyglycine. The genetic code evolved as described in this report. Primitive pre-mRNAs and pre-rRNAs were generated by similar processes of ligation and genetic recombination.

To evolve tRNA required a small number of catalytic functions (i.e., ribozymes). The process required a mechanism to generate RNA repeats and inverted repeats. Multiple functions were necessary, including RNA ligase, RNA replicase (complementary replication), exo- and endo-nucleases, ribose 2′-O-methyltransferase (for RNA stability) and ACCA-Gly transferase. Complementary replication utilizing snap-back primers (i.e., 31 nt minihelices) was needed. With these ingredients and little else, it should be possible to recreate most of the origin of tRNA and the genetic code in a laboratory. The evolution of tRNA and the genetic code describe an RNA–amino acid and RNA–peptide world overlaid on primitive metabolism with coevolution of protocells to generate the first life on Earth.

## 8. Discussion

The genetic code coevolved with tRNA, tRNAomes, AARSomes, ribosomes and the first proteins [5,6,7,8,9,10]. The evolution of AARSomes is evident in genetic code columns. In column 1, ValRS-IA, LeuRS-IA, IleRS-IA and MetRS-IA are closely related enzymes. In column 2, SerRS-IIA, ProRS-IIA and ThrRS-IIA are closely related enzymes. AlaRS-IID may have replaced a now extinct AlaRS-IIA before LUCA. Column 3 demonstrates a striped pattern of related AARS enzymes. AspRS-IIB, AsnRS-IIB and HisRS-IIA are closely related enzymes in rows 4A, 3A and 2A. GluRS-IB, LysRS-IB (in Archaea) and GlnRS-IB (a eukaryotic innovation) are closely related enzymes in rows 4B, 3B and 2B. A primitive GlyRS-IIA appears to be the founding AARS. tRNA^Gly^ appears to be the founding tRNA that is most similar to tRNA^Pri^ [21]. Glycine appears to be the founding amino acid [19,20], and glycine occupies the most favored sector in the code (tRNA-35C, tRNA-36C). In column 4, ArgRS-IA and CysRS-IA are closely related enzymes. Row 1 of the genetic code appears to have been sectored last. TrpRS-IC and TyrRS-IC are closely related enzymes. PheRS-IIC appears to be a late substitution, perhaps for a PheRS-IC, from which TyrRS-IC and TrpRS-IC were derived. According to this view, the hypothesized PheRS-IC is now extinct. Cysteine may have first entered the code through tRNA-linked chemistry within an expanded serine sector.

Coding evolved around tRNA and the tRNA anticodon. Coding should be viewed as arising first in the tRNA anticodon. In tRNA, the maximum number of coding assignments is limited to 32 by wobbling. tRNA cannot support 64 genetic code assignments, as can DNA and mRNA. Coding coevolved from tRNA anticodons into mRNA codons and then was cast into DNA for more stable information storage. Degeneracy of the code is a feature of tRNA and the tRNA anticodon. Wobbling at tRNA-34 created code degeneracy. The suppression of wobbling at tRNA-36 gives the history of genetic code establishment. Even with modifications, no tRNA-34A is utilized at the base of the genetic code evolution. Elp3 and subsequent tRNA-34U 5-carbon modifications suppressed superwobbling in order to evolve 2-codon sectors in the code (i.e., column 3) [61,62,63]. tRNA-34, tRNA-37 and other tRNA modifications were necessary to evolve the first code.

Because of the placement of the anticodon loop U-turn, wobbling at tRNA-36 was suppressed, but wobbling at tRNA-34 was not. Next to tRNA-34 is tRNA-33U, which is on the opposite side of the anticodon U-turn. Because of the placement of the U-turn, modifying tRNA-33U would be unlikely to influence reading at tRNA-34. Also, tRNA-33U is almost never substituted, indicating that a purine at that position might disrupt loop geometry. Modifications of tRNA-35 cannot compensate because 35 is a Watson–Crick position for coding that cannot be specified in sequence or modified in a manner that affects coding. Apparently, modifications of tRNA-37 helped to suppress wobbling at tRNA-36, particularly for tRNA-36U (i.e., tRNA-37t6A) and tRNA-36A (i.e., tRNA-37m1G) [61]. To evolve the first code, these modifications may have been universal. As systems have evolved, some compensations for some modifications may have coevolved. Wobbling at tRNA-34 (regulated) versus tRNA-36 (suppressed) appears to explain why columns 1, 2 and 4 differ in their sectoring from column 3, which is the most innovated column.

## 9. Conclusions

The genetic code is simpler in Archaea than in Bacteria and Eukarya, indicating that the archaeal code is most similar to the LUCA code. The code in Archaea is highly ordered, and the order provides the history for first code establishment. tRNAomes are simpler in Archaea. Organisms with the simplest tRNAomes are the closest to LUCA. tRNAome and AARSome networks of ancient organisms describe the history of the establishment of the first code.

tRNA evolved from RNA repeats and inverted repeats of known sequence. Three 31 nt minihelices were ligated and processed by orderly internal 9 nt deletion(s) into type I and type II tRNAs [15,31,120] (Figure 1 and Figure 2). In pre-life, multiple RNAs were joined as replication intermediates, generating long functional RNAs, such as tRNAs, pre-mRNAs and primitive rRNAs. tRNA evolution is a story of amino acid–RNA and protein–RNA-linked chemistry [43], so life evolved from a complex RNA–amino acid–RNA–protein–metabolism world, packaged in coevolved protocells. When coupled with coded protein synthesis, this evolving pre-life world generated remarkable complexity and fostered surprising innovation. The first proteins that coevolved with the genetic code were highly evolved, innovated and complex constructs, many of which remain largely unaltered to the present day. With the freezing of the first code, life as currently known emerged on Earth. The history of tRNA evolution is embedded in tRNA sequences, which can be read. The history of the evolution of the genetic code is embedded in code structure and interacting tRNAome, AARSome and first protein networks.

The core history of abiogenesis is the evolution of tRNA, which was recorded and preserved in tRNA sequences. The history of genetic code evolution was written into the standard genetic code structure and AARSome radiation. AARSome structure provides a history that describes genetic code structure and evolution. tRNAome structure contributed to genetic code assembly but was less important for directing the structure of the code.

Based on tRNA sequences, we posit that RNA repeat and inverted repeat worlds gave way to a 31 nt minihelix world, which evolved to a tRNA world (Figure 1 and Figure 2). Thus, peptide synthesis pre-dated tRNA, and tRNA evolved from 31 nt minihelices as an improved mechanism to synthesize long peptides (i.e., polyglycine). tRNA evolved from a world that was capable of complex metabolism and RNA sequence manipulation (i.e., the selection and ordered assembly of short, stable RNA stems and 7 nt U-turn loops). Polyglycine and polyGADV amyloids and polymers were an important feature of emerging life and the evolving code. Cells were emulsified from the inside by polypeptide polymers, coacervates and amyloids and from the outside by membrane encapsulation.

For astrobiology, because of the challenges generating a genetic adapter and a code, it is difficult to see how life could evolve separately on another planet or moon by a very different chemistry or different pathway. If there is another route to a suitable genetic adapter than tRNA, we are not certain what that might be. Life without a genetic adapter and genetic code has limited possibilities.

## Data Availability

No new data were created or analyzed in this study.

## Abbreviations

The following abbreviations are used in this manuscript:

AARS	Aminoacyl-tRNA synthetase
Aae	*Aquifex aeolicus*
Bta	*Bos taurus*
Eco	*Escherichia coli*
Hvo	*Haloferax volcanii*
Hsa	*Homo sapiens*
Lbp	Levitt base pair
Lla	*Lactobacillus lactis*
LUCA	Last universal common (cellular) ancestor
Mca	*Mycoplasma capricolum*
Pri	Primordial
Pfu	*Pyrococcus furiosus*
Sau	*Staphylococcus aureus*
Sgr	*Streptomyces griseus*
Tac	*Thermoplasma acidophilum*
Tth	*Thermus thermophilus*
